# Omics Insights into Cylindrospermopsin’s Molecular Toxicity

**DOI:** 10.3390/foods14213620

**Published:** 2025-10-23

**Authors:** Ronald F. Borja, Cristina Plata-Calzado, Leticia Diez-Quijada, María Puerto

**Affiliations:** Área de Toxicología, Facultad de Farmacia, Universidad de Sevilla, C/Profesor García González Nº 2, 41012 Seville, Spain; rborja1@us.es (R.F.B.); cpcalzado@us.es (C.P.-C.); ldiezquijada@us.es (L.D.-Q.)

**Keywords:** Cylindrospermopsin, omics, toxicity, in vitro, in vivo, food safety

## Abstract

Cylindrospermopsin (CYN) is a potent cyanotoxin that poses a significant risk to human and animal health. Due to its occurrence in drinking water and food, as well as its ability to bioaccumulate in aquatic organisms and plants irrigated with contaminated water, the oral route is an important exposure pathway. However, data gaps in the current toxicological data for CYN jeopardize the establishment of health guidance values. In this context, mechanistic data and a deeper knowledge of CYN’s mode of action and its adverse outcome pathways are priorities for risk assessment. In recent years, omics techniques have enabled important advances in the comprehensive characterization of the molecular toxicity of CYN. In vitro studies have mainly focused on liver and kidney models, while in vivo studies have mostly used aquatic organisms. These studies have shown effects at both the transcriptional and protein levels on various signaling pathways related to detoxification, DNA damage, apoptosis, cell survival, and lipid metabolism, among others. However, studies using lipidomic, metabolomic, or microbiomic techniques are limited to date. Nevertheless, a recent study suggests that CYN may also induce gut dysbiosis, which would further extend its toxicological profile. This review emphasizes the need to further expand the use of omics approaches to accurately assess the risks associated with the consumption of CYN-contaminated foods.

## 1. Introduction

Cyanobacteria are prokaryotic microorganisms commonly found in marine and freshwater environments, where they can form massive proliferations known as cyanobacterial blooms. These blooms have increased worldwide due to anthropogenic activities, eutrophication, and global climate change [1,2], raising serious concerns owing to their ability to produce secondary metabolites known as cyanotoxins. These compounds can have serious ecological and health impacts, including damage to fisheries and agricultural systems and degradation of water quality for human consumption and recreational activities [3]. Human exposure to cyanotoxins has been linked to various health effects ranging from mild symptoms, such as skin irritation and gastroenteritis, to severe outcomes, including liver damage, renal failure, and, in extreme cases, even death [2,4].

Among cyanotoxins, Cylindrospermopsin (CYN) is the second most studied after Microcystins (MCs) and has gained increasing attention due to its global distribution, bioaccumulation, and multi-organ toxicity [5,6]. CYN, originally isolated from the cyanobacterium *Raphidiopsis raciborskii*, is an alkaloid (C_15_H_21_N_5_O_7_S; 415.43 Da) with a tricyclic guanidine moiety, a sulfate group, and an uracil ring [7]. To date, eight naturally occurring variants of CYN have been identified, including 7-deoxydesulfo-CYN, 7-deoxy-CYN, 7-Epi-CYN, and 7-deoxydesulfo-12-acetyl-CYN [2]. CYN principally targets the liver and kidney, but it can also affect other organs, including the thymus, heart, spleen, lungs, and eyes. Furthermore, CYN presents a wide range of toxic effects, including cytotoxic, genotoxic, immunotoxic, neurotoxic, endocrine, and developmental effects. Its main mechanism of action is the inhibition of protein synthesis; however, it can also interact with cytochrome P450 (CYP450) enzymes, induce oxidative stress and DNA fragmentation, form micronuclei, produce immunomodulatory effects, interfere with acetylcholinesterase (AChE) activity, and display antagonistic effects on the estrogen receptors [6,8,9,10,11].

Human exposure to CYN occurs mainly through ingestion of contaminated drinking water, although inhalation or dermal exposure during recreational activities such as swimming, bathing, or water skiing may also be significant routes of exposure [12,13]. Moreover, food can also be a significant pathway of exposure due to the potential bioaccumulation of CYN. In this regard, vegetables may pose a risk, particularly when grown near bodies of water affected by cyanobacterial blooms [12] or when contaminated water is used for irrigation [14], as several studies have shown that edible vegetables, such as lettuce, arugula [14], or spinach [15], are capable of accumulating CYN. Additionally, this cyanotoxin can bioaccumulate in aquatic organisms such as fish, mollusks, and crustaceans, which are, afterwards, consumed by humans and are, subsequently, introduced into the trophic chain [16]. Indeed, various studies have confirmed the presence of CYN in the tissues of aquatic organisms at concentrations up to 4.3 µg/g and have suggested bioaccumulation factors of CYN between 4 and 171, highlighting the toxin’s potential to bioaccumulate in aquatic species [3]. Notably, CYN can also persist through some conventional water treatment processes, further increasing its potential for human exposure through drinking water and food [17].

Given these risks, the World Health Organization (WHO) has established provisional guideline values to limit human exposure. These include a lifetime drinking-water guideline value of 0.7 µg/L, a short-term drinking-water guideline of 3 µg/L, a recreational water guideline of 6 µg/L, and a tolerable daily intake (TDI) of 0.03 µg/kg of body weight (bw) per day [18]. However, these values remain provisional due to gaps in toxicological data, mechanisms of action, chronic effects, and long-term risks. These data gaps were also identified by the European Food Safety Authority (EFSA), which states that TDI should be updated once genotoxic and/or carcinogenic properties of this cyanotoxin are demonstrated [19]. In this context, omics-based techniques could help reduce the uncertainty surrounding CYN in relation to provisional guideline values and improve public health protection.

Omics technologies have made incredible strides in the last ten years, such that in toxicology research, they are powerful tools for generating accurate and relevant data on the molecular changes that lead to adverse health effects [20]. Compared to older methods for measuring toxicant-induced molecular alterations, omics technologies offer a promising path to improve chemical safety assessments and potentially reduce the need for animal testing [21], and, therefore, they contribute to the Next Generation Risk Assessment. These technologies’ approaches encompass different fields, including genomics (analysis of the full genetic material of an organism), transcriptomics (study of gene expression by RNA), proteomics (profiling of protein expression), and metabolomics (identification and quantification of metabolites such as amino acids, lipids, or carbohydrates). Other relevant omics include microbiomics, epigenomics, and more specialized subfields like lipidomics, a subset of metabolomics focused specifically on lipid profiles [22,23]. Importantly, each omics captures distinct biological information, making multi-omics integration a particularly valuable strategy. By combining datasets from multiple omics platforms, researchers can obtain a more holistic view of CYN-induced toxicity, from molecular initiating events to organism-level effects. In this context, the integration of omics technologies into the food sector has given rise to foodomics. This multidisciplinary field plays a crucial role in ensuring food quality and safety, enabling a comprehensive analysis of food products and the biological systems to which they are exposed. Foodomics uses high-throughput omic methodologies, such as those previously described, for the early, rapid, and reliable detection of potential hazards, including adulterants, pathogens, and toxins. In addition, this emerging discipline has further evolved by incorporating biostatistics, chemometrics, and bioinformatics, offering a more robust and integrated framework for food safety assessment [24,25]. Specifically, metabolomics is a very effective tool for safety control, as it can directly describe the metabolic response of a biological system to contaminants. In fact, metabolomics is widely used for safety control in complex matrices [26]. This context strengthens the relevance of applying foodomics to assess and mitigate the potential risks posed by CYN contamination within the food system.

Therefore, the aim of this review is to provide a comprehensive revision and summary of the current state of knowledge on the toxicological profile and effects of CYN investigated through omics techniques. By analyzing studies that employ transcriptomics, proteomics, metabolomics, and other omics approaches, this work will highlight the key molecular responses to CYN exposure in different species and tissues, consequently, advancing the comprehension of CYN’s mechanisms of toxicity.

## 2. Literature Search Strategy

A structured literature search was conducted to ensure a comprehensive and current review of the toxicological effects and molecular mechanisms of CYN. Primary databases included Web of Science, Scopus, PubMed, and Google Scholar. The search was supplemented by reviewing the bibliographies of relevant articles, and additional sources were identified through a cross-referencing key. Publications considered were those from 2007 to July 2025.

Keywords used individually and in combination with Boolean operators (AND, OR) included: “cylindrospermopsin,” “cyanotoxins,” “toxicity,” “omics,” “transcriptomics,” “proteomics,” “metabolomics,” “microbiome,” “hepatotoxicity,” “neurotoxicity,” “in vitro,” and “in vivo.” This approach was designed to cover a wide spectrum of studies on CYN toxicity, from molecular mechanisms to cellular and organismal effects. In addition, the bibliography of these articles has been reviewed to complete the search.

This search strategy ensures a comprehensive and up-to-date review of the literature on CYN, integrating toxicological research across various omics approaches and in both in vitro and in vivo models.

### Eligibility and Exclusion Criteria

The following criteria were considered in the information selection process:

Inclusion criteria: (1) articles on the toxicological effects and molecular mechanisms of CYN in vitro; (2) articles on the toxicological effects and molecular mechanisms of CYN in vivo; (3) articles published between 2007 and July 2025; and (4) articles reporting comprehensive results published in internationally recognized journals.

Exclusion criteria: (1) articles on CYN toxicity that do not explore molecular mechanisms; (2) articles published in a language other than English; (3) proceedings of conferences and dissertations; and (4) where the abstracts were only available.

## 3. The Role of Omics in Understanding Cylindrospermopsin

### 3.1. Transcriptomics

#### 3.1.1. Transcriptomic Studies on CYN-Mediated Liver Toxicity

Transcriptomic analyses of CYN have primarily focused on human hepatic cells (Table 1), particularly in the HepG2 cell line, with research largely centered on mechanisms of detoxification response, DNA damage, and immediate–early response/signaling [27,28,29,30,31,32]. Multiple studies have consistently shown a time-dependent up-regulation of CYP450 enzymes, particularly CYP1A1 (cytochrome P450, family 1, subfamily A, polypeptide 1) and CYP1A2 (cytochrome P450, family 1, subfamily A, polypeptide 2) isoforms following CYN exposure [27,29,30,31,32]. Moreover, transcriptional analyses indicate the involvement of phase II enzymes in CYN detoxification [32]. Regarding DNA damage response, the expression of *TP53* (tumor protein P53) was unaffected (0–0.5 µg/mL) by CYN [27,29,31,32] or slightly down-regulated [28,33]. This suggests that CYN’s primary impact may involve genes downstream of p53, such as *GADD45* (growth arrest and DNA-damage-inducible) and *CDKN1A* (cyclin-dependent kinase inhibitor 1A), which are indeed related to p53 signaling and cell cycle regulation [27,28,29,30,31,32]. In these same cells, Straser et al. [32] demonstrated that CYN exposure triggered a significant upregulation of immediate–early response genes, with expression levels increasing more than fourfold. In particular, *FOS* (FBJ murine osteosarcoma viral oncogene homolog), *FOSB* (FBJ murine osteosarcoma viral oncogene homolog B), and *JUNB* (Jun B proto-oncogene) were significantly up-regulated after 12 h of treatment and further enhanced after 24 h of exposure. Additionally, apoptosis/survival pathways have also been investigated in these cells [27,28,29,30,31,32], showing that CYN did not cause severe perturbations. While several up-regulated genes suggest the activation of both extrinsic and intrinsic apoptotic pathways, the observed deregulation pattern of BCL2 family genes and several other factors ultimately favors apoptosis suppression [32].

Niture et al. [34] recently demonstrated that exposing liver cell models (HepG2 and SK-Hep1cells) to 250 nM CYN for 72 h modulated the expression of pro-inflammatory genes. Given that the unfolded protein response (UPR) is a cellular protective mechanism activated by endoplasmic reticulum (ER) stress, and that sustained ER stress and subsequent UPR activation have been linked to cell death [35], this study showed the impact of CYN on the regulation of UPR gene biomarkers using reverse transcription quantitative polymerase chain reaction (RT-qPCR). Furthermore, CYN decreased lipogenic gene expression in both cell lines. Importantly, RNA sequencing data suggested that CYN induced the expression of genes associated with non-alcoholic fatty liver disease (NAFLD), potentially promoting its development and progression in human hepatocytes [34].

Vanova et al. [36] studied the effect of CYN on hepatic differentiation from human embryonic stem cells (hESCs). They observed that early-stage hepatic markers like *FOXA2* (Forkhead box A2), *AFP* (Alpha Fetoprotein), and *CX43* (Gap Junction Protein Alpha 1) remained relatively unchanged, whereas later-stage markers such as *ALB* (albumin), *TTR* (transthyretin), and *CX32* (Gap Junction Protein Beta 1) showed reduced expression. Furthermore, CYN’s effects on hepatocyte-specific functions, including ALB secretion and lipid droplet accumulation, were more pronounced in immature (D15) than in mature (D20) hepatocytes. The observed decrease in ALB secretion following CYN treatment correlates with reduced ALB expression, suggesting that the elimination of ALB-producing cells is a contributing factor. Huguet et al.’s [37] transcriptomic study using differentiated HepaRG cells revealed that CYN exposure upregulated genes involved in RNA maturation, suggesting that CYN modified the expression of proteins involved in RNA modification and maturation prior to translation. Simultaneously, genes involved in xenobiotic metabolism and cell cycle progression were down-regulated. The 1055 down-regulated genes were associated with 64 biological processes (including 23 related to mitosis and 18 involved in cellular metabolism of lipids, alcohols, and organic acids), 38 cellular components (primarily microtubules and chromosomes, and secondarily vesicles, lysosomes, and the endoplasmic reticulum), and 25 molecular functions (mainly oxidoreductase and transferase enzyme activities).

On the other hand, the effects of CYN on liver stem cells (LSCs), which are crucial for liver tissue development, regeneration, and repair, are poorly understood. Raska et al. [38] found that while CYN did not affect *HSPA5* (Heat Shock Protein Family A (Hsp70) Member 5, an ER stress/UPR marker), it significantly increased *ATF3* (Activating Transcription Factor 3, a highly versatile stress sensor for liver injury) expression in LSCs between 24 and 48 h after exposure by RT-PCR.

Moreover, transcriptomic analyses using hepatic 3D cell models to assess the effects of CYN have been conducted [30,39]. Unlike traditional 2D cultures, these 3D liver cell models exhibit enhanced cell–cell and cell–matrix interactions, forming more realistic tissue-like structures. Crucially, 3D models improve metabolic activity and liver function, resulting in more in vivo-like responses in terms of cell viability, proliferation, differentiation, morphology, gene and protein expression, and overall cellular function [40]. The analysis of gene expression in HepG2 3D cells exposed to CYN revelated that this toxin affects the transcription of genes encoding phase I and phase II enzymes, with a strong up-regulation of *CYP1A2* (13.2 fold) and all studied phase II enzymes, including *NAT1* (N-Acetyltransferase 1), *NAT2* (N-Acetyltransferase 2), *SULT1B1* (Sulfotransferase Family 1B Member 1), *SULT1C2* (Sulfotransferase Family 1C Member 2), *UGT1A1* (UDP Glucuronosyltransferase Family 1 Member A1), and *UGT2B7* (UDP Glucuronosyltransferase Family 2 Member B7). This indicates that the expression of metabolic genes, especially those involved in detoxification (phase II), was higher than previously reported in 2D cultures. Moreover, CYN exposure in HepG2 spheroids induced DNA damage, evidenced by the up-regulation of DNA damage response genes and cell cycle arrest. It also becomes evident that CYN’s mechanisms of action involve the induction of apoptosis (BCL2 Binding Component 3, *BBC3*), the lipogenesis (*SREBF1*, encoding sterol regulatory element-binding protein 1), and the acylglycerol synthesis (diacylglycerol O-acyltransferase 1/2, *DGAT1/2*). Conversely, CYN decreased fatty acid synthesis gene expression, while fatty acid uptake, oxidation, and lipid efflux gene expression remained unchanged [30,39].

Regarding in vivo experimental models, the expression of gene markers involved in lipid metabolism and oxidative stress is among the most frequently studied [41,42,43,44,45,46,47].

Chernoff et al. [41] demonstrated that daily intraperitoneal (i.p.) injections of 50 µg CYN/kg in pregnant mice, administered over seven weeks of gestation, altered the expression profiles of genes across several crucial pathways. Microarray analysis revealed impacts not only on lipid metabolism but also on ribosomal biogenesis, xenobiotic and inflammatory responses, and the Nrf2 (Nuclear factor erythroid 2-related factor 2) oxidative stress response. The lack of discernible differences in gene expression between dose groups further supported the hypothesis that liver injury may not be the primary mechanism of CYN-induced toxicity in mice. In a separate 90-day study, oral CYN exposure (75 to 300 µg/kg/day) in mice affected the expression of genes implicated in liver regeneration, pancreatic disease, and apoptosis. The expression of genes related to blood coagulation and the fatty acid-binding protein 4 (*Fabp4*) gene, which is implicated in fatty acid uptake and metabolism, was significantly down-regulated at all dose levels, except in high-dose males [42].

Gene expression was recently evaluated in tumor tissue from 16 hepatocellular carcinoma patients with available tumor samples. Moreover, cyanotoxin levels were measured in serum by ELISA. The study compared cyanotoxin levels with the tumor expression of over 700 genes (using a Nanostring nCounter Fibrosis panel, Seattle, WA, USA) [46]. CYN levels varied significantly based on the underlying cause of cancer, with the highest levels observed in patients with metabolic risk factors such as hyperlipidemia, type 2 diabetes, and non-alcoholic fatty liver disease/non-alcoholic steatohepatitis (NAFLD/NASH). Furthermore, a significant positive correlation was found between CYN levels and the tumor expression of genes involved in peroxisome proliferator-activated receptor (*PPAR*) signaling and lipid metabolism.

On the other hand, oxidative stress at the transcriptional level has been primarily studied in tilapia (*Oreochromis niloticus*) [44,45,47]. These studies focused on the effects of CYN exposure (200 and 400 µg/mL) on the transcription of glutathione peroxidase (*Gpx*) and glutathione-S-transferase (*Gst*) in relation to the exposure route (oral, gavage, or i.p. injection) and the time of sacrifice (24 h or 5 days). The results demonstrated that both conditions (exposure route and time) significantly influenced the observed transcriptomic effects. Five days post-exposure yielded the most pronounced changes, particularly the up-regulation of *Gpx* and *Gst*, primarily following i.p. injection [45,47]. Gutiérrez-Praena et al. [44] also demonstrated that both pure CYN and CYN from lyophilized cell cultures affected the gene expression of these markers.

Overall, transcriptomic studies highlight the liver as a primary target of CYN toxicity, affecting detoxification pathways, DNA damage response, lipid metabolism, and oxidative stress. Moreover, analyses indicate that CYN can disrupt hepatic differentiation, modulate phase I and II enzyme expression, and alter genes associated with NAFLD.

**Table 1:** Transcriptomic Analysis of CYN Toxicity: Liver.

Experimental Model	Exposure Concentration/Doses	Exposure Time	Route	Omics Type	Omics Technique	Pathway and Genes Analyzed	Reference
HepG2 cells	1, 2.5, 5 µg/mL	6 h	In vitro	Transcriptomics	qRT-PCR	DNA damage: *CDKN1A*, *GADD45a*, *MDM2* Apoptosis/survival: *BAX*	[27]
	1 µg/mL	24 h					
HepG2 cells	0.005, 0.05, 0.5 µg/mL	4, 12, 24 h	In vitro	Transcriptomics	qRT-PCR	Metabolism: *CYP1A1* and *CYP1A2* DNA damage: *P53*, *CDKN1A*, *GADD45*, *MDM2*	[31]
HepG2 cells	0.5 µg/mL	12, 24 h	In vitro	Transcriptomics	qPCR-arrays	Immediate–early response/signaling: *FOSB*, *FOS*, *JUNB*, *TGFB2*, *JUN*, *GDF15*, *NFKB1*, *GAB1* Metabolism: *CAT*, *ALDH1A2*, *CYP1A1*, *CYP1B1*, *UGT1*, *TXNRD1*, *NAT1*, *GCLC*, *CES2*, *GSTM3*, *UGT1A1*, *CYP2A6*, *CYP2A13*, *CYP3A43*, *CYP3A7*, *GSTM2*, *CYP2F1*, *GSTA2*, *CES1*, *GNMT*, *SULT1A1* Cell cycle/proliferation: *GADD45B*, *GADD45A*, *CDKN1A*, *CDKN2B*, *HUS1*, *CHEK1*, *CDK7*, *CCNE2*, *E2F4*, *PCNA*, *CDKN2C*, *CCNG1*, *TFDP1*, *RAD1* DNA damage repair: *XPC*, *ERCC4*, *LIG4*, *MSH3*, *XRCC2*, *RAD51*, *MRE11A*, *MRE11*, *BRCA2*, *POLB* Apoptosis/survival: *FAS*, *DIABLO*, *TIMP1*, *BCL2L1*, *TNF*, *TNFAIP3*, *TNFRSF10A*, *MCL1*, *CASP9*, *BAK1*, *TRADD*, *CASP3*, *FOXO3*, *CASP8*, *DDIT3*, *BCL2*, *TNFSF10*, *BID*, *APAF1*, *CASP7*	[32]
HepG2 cells	0.01, 0.5 µg/mL	4, 24 h	In vitro	Transcriptomics	qRT-PCR	Immediate–early response/signaling: *FOS*, *JUNB*, *MYC*, *TGFB2* Metabolism: *CYP1A1*, *CYP1A2*, *CYP3A4*, *CYP1B1*, *NAT*, *GSTA1*, *UGT1A1* DNA damage: *TP53*, *MDM2*, *CDKN1A*, *GADD45A*, *CHEK1*, *ERCC4*	[29]
HepG2 cells	0.5 µg/mL /bisphenol A	24 h	In vitro	Transcriptomics	qRT-PCR	Metabolism: *CYP1A1*, *GCLC* DNA damage: *P53*, *CDKN1A*, *GADD45*, *MDM2*, *CHEK1* Oxidative stress: *GPX1*, *GR*, *SOD1A*, *CAT*	[33]
HepG2 cells	0.5 µg/mL CYN 1 µg/mL MC-LR 0.5 µg/mL CYN + 1 µg/mL MC-LR	72 h	In vitro	Transcriptomics	qRT-PCR	Immediate–early response/signaling: *JUNB* Metabolism: *CYP1A1*, *UGT1A1* DNA damage: *TP53*, *MDM2*, *CDKN1A*, *GADD45A*	[28]
HepG2 SK-Hep1 cells Hepatocytes HepG2 HepaRG	250 nM	72 h	In vitro	Transcriptomics	RT-qPCR	Pro-inflammatory: *IL-6*, *TNF-α*, *TNFAIP8* UPR: *IRE1a*, *eIF2a*, *ATF4*, *ATF6*, and *BIP* Lipogenic genes: *SREPB1*, *FABP1*, *SCD1*, *FASN*, *ACC*, *PPAR-α* Fibrosis: *TIMP2*, *TGFB1*, *FGF-23*, *CX3CR* *NAFLD* development and progression	[34]
					RNA-Seq		
Hepatic differentiation from human embryonic stem cells	1 µM	48 h	In vitro	Transcriptomics	PCR	Apoptosis/survival: *FOXA2*, *HNF4A*, *AFP*, *TTR*, *ALB*, Fibrosis: *CX42* and *CX32*	[36]
Differentiated HepaRG cells	0.8 µM	24 h	In vitro	Transcriptomics	microarray	Modified the expression of genes involved in metabolism, RNA processing, cell cycle	[37]
Liver stem cells	1 µM	24, 48 h	In vitro	Transcriptomics	RT-PCR	UPR: *ATF3*, *HSPA5*	[38]
HepG2 3D cell spheroids	0.125, 0.25, 0.5 µg/mL	72 h	In vitro	Transcriptomics	RT-qPCR	Metabolism: *CYP1A1*, *CYP1A2*, *CYP3A4; ALDH3A1; AHR*, *NAT1*, *NAT2*, *SULT1B1*, *SULT1C2*, *UGT1A1*, *UGT2B7* Oxidative stress: *H1F1A* DNA damage: *CDKN1A*, *GADD45*, *ERCC4* Cell cycle arrest: *CCND1* Apoptosis/survival: *BBC3* Cell cycle/proliferation: *PCNA*, *TOP2α*, *MK167*	[30]
HepG2 3D cell spheroids	1 µM	48 h	In vitro	Transcriptomics	RT-PCR	Fatty acid synthesis genes: *ACLY*, *ACCA*, *FASN*, *SCD1* Triacylglycerol synthesis genes: *DGAT1*, *DGAT2* lipogenesis-regulating gene: *SREBF1* Fatty acid uptake: *FAT/CD36* fatty acid Oxidation: *CPT1A* lipid efflux genes: *APOB*	[39]
Zebrafish (*Danio rerio*)	20 µg/L CYN	14 d	Waterborne exposure	Transcriptomics	RT-PCR	Metabolism: *Cyp1a*, *Cyp26*, *Ephx1* DNA damage detection and repair: *Gadd45*, *Rad51*, *Jun*, *Xpc* Apoptosis/survival: *Caspase 3a* and *3b*, *Bcl-2*, *Bax*, *p53*, *Mapk*, *Nrf2* Lipid metabolism: *Ppara*, *Fabp1*, *Pla2* Phosphorylation/dephosphorylation: *Ppp6c*, *Ppm1* Cytoskeleton: *actin*, *tubulin*	[43]
Tilapia (*Oreochromis niloticus*)	200–400 µg/kg CYN	24 h	Gavage	Transcriptomics	RT-PCR	Oxidative stress: *Gpx*, *Gst*	[47]
Tilapia (*Oreochromis niloticus*)	Pure and Lyoph 200 µg/mL + (NAC)	24 h	Oral	Transcriptomics	RT-PCR	Oxidative stress: *Gpx*, *Gst*	[44]
Tilapia (*Oreochromis niloticus*)	200 µg/mL	24 h, 5 d	Gavage i.p. injection	Transcriptomics	RT-PCR	Oxidative stress: *Gpx*, *Gst*	[45]
Pregnant mice	50 µg/kg	7 weeks (gestational periods)	i.p. injection	Transcriptomics	Microarray	Lipid metabolism, ribosomal biogenesis, metabolism, inflammatory responses and oxidative stress.	[41]
					RT-PCR		
Mice	75–300 µg/kg/day	90 d	Oral	Transcriptomics	RT-PCR	Liver regeneration: *Rpl6* Pancreatic disease: *Nupr1* Apoptosis/survival: *Bax*, *Trp53*, *c-Jun* Blood coagulation: *Proc. Klkb1*, *Thbs1*, *Thpo* Fatty acid metabolism: *Fabp4*	[42]
Liver cancer patients	Tumor tissue	(Oral)	In vivo	Transcriptomics	Nanostring nCounter	CYN detected in sera of all patients 700 genes analyzed in tissue mainly correlated with tumor expression of genes functioning in PPAR signaling and lipid metabolism	[46]

Abbreviations: *ACC*: Acetyl-CoA Carboxylase; *ACCA*: Acetyl-CoA Carboxylase A; *ACLY*: ATP Citrate Lyase; *AFP*: Alpha Fetoprotein; *AHR*: Aryl Hydrocarbon Receptor; *ALDH1A2*: Aldehyde dehydrogenase 1 family, member A2; *ALDH3A1*: Aldehyde Dehydrogenase 3 Family 1; *APAF1*: Apoptotic peptidase activating factor 1; *APOB*: Apolipoprotein B; *ATF4*: Activating Transcription Factor 4; *ATF6*: Activating Transcription Factor 6; *BAK1*: BCL2-antagonist/killer 1; *BAX*: BCL2-Associated X Protein; *BBC3*: BCL2-Binding Component 3; *BCL2*: B-cell CLL/lymphoma 2; *BCL2L1*: BCL2-like 1; *BID*: BH3 interacting domain death agonist; *BIP*: Binding Immunoglobulin Protein; *BRCA2*: Breast cancer 2, early onset; c-Jun: Jun proto-oncogene; *CASP3*: Caspase 3, apoptosis-related cysteine peptidase; *CASP7*: Caspase 7, apoptosis-related cysteine peptidase; *CASP8*: Caspase 8, apoptosis-related cysteine peptidase; *CASP9*: Caspase 9, apoptosis-related cysteine peptidase; *CAT*: Catalase; *CCND1*: Cyclin D1; *CCNE2*: Cyclin E2; *CCNG1*: Cyclin G1; *CDK7*: Cyclin-dependent kinase 7; *CDKN1A*: Cyclin-Dependent Kinase Inhibitor 1A; *CDKN2B*: Cyclin-dependent kinase inhibitor 2B; *CDKN2C*: Cyclin-dependent kinase inhibitor 2C; *CES1*: Carboxylesterase 1; *CES2*: Carboxylesterase 2; *CHEK1*: Checkpoint kinase 1; *CPT1A*: Carnitine Palmitoyltransferase 1A; CX32: Gap Junction Protein Beta; 1CX3CR: CX3C Chemokine Receptor; CYP1A: cytochrome P450, family 1, subfamily A; *CYP1A1*: cytochrome P450, family 1, subfamily A, polypeptide 1; *CYP1A2*: cytochrome P450, family 1, subfamily A, polypeptide 2; *CYP1B1*: Cytochrome P450, family 1, subfamily B, polypeptide 1; *CYP26*: Cytochrome P450 26; *CYP2A13*: Cytochrome P450, family 2, subfamily A, polypeptide 6; *CYP2A6*: Cytochrome P450, family 2, subfamily A, polypeptide 6; *CYP2F1*: Cytochrome P450, family 2, subfamily F, polypeptide 1; *CYP3A4*: cytochrome P450 family 3 subfamily A member 4; *CYP3A43*: Cytochrome P450, family 3, subfamily A, polypeptide 43; *CYP3A7*: Cytochrome P450, family 3, subfamily A, polypeptide 7; *DDIT3*: DNA-damage-inducible transcript 3; *DGAT1*: Diacylglycerol Acyltransferase 1; *DGAT2*: Diacylglycerol Acyltransferase 2; *DIABLO*: Diablo, IAP-binding mitochondrial protein; *E2F4*: E2F transcription factor 4, p107/p130-binding; eIF2a: Eukaryotic Translation Initiation Factor 2 alpha; *EPHX1*: Epoxide Hydrolase 1; *ERCC4*: Excision repair cross-complementing rodent repair deficiency, complementation group 4; *FABP1*: Fatty Acid Binding Protein 1; Fabp4: Fatty Acid Binding Protein 4; *FAS*: Fas (TNF receptor superfamily, member 6); *FASN*: Fatty Acid Synthase; *FAT/CD36*: Fatty Acid Translocase/Cluster of Differentiation 36; *FGF-23*: Fibroblast Growth Factor 23; *FOS*: FBJ murine osteosarcoma viral oncogene homolog; *FOSB*: FBJ murine osteosarcoma viral oncogene homolog B; *FOXA2*: Forkhead box A2; *FOXO3*: Forkhead box O3; *GAB1*: GRB2-associated binding protein 1; *GADD45A*: Growth arrest and DNA-damage-inducible, Alpha; *GADD45B*: Growth arrest and DNA-damage-inducible, Beta; *GCLC*: Glutamate-cysteine ligase, catalytic subunit; *GDF15*: growth differentiation factor 15; *GNMT*: Glycine N-methyltransferase; *GPx*: Glutathione Peroxidase; *GR*: Glutathione reductase; *GST*: Glutathione S-Transferase; *GSTA1*: glutathione S-transferase alpha 1; *GSTA2*: Glutathione S-transferase alpha 2; *GSTM2*: Glutathione S-transferase mu 2 (muscle); *GSTM3*: Glutathione S-transferase mu 3 (brain); H1F1A: Hypoxia Inducible Factor 1 Alpha; HSPA5: Heat Shock Protein Family A (Hsp70) Member 5; HUS1: HUS1 checkpoint homolog (*S. pombe*); IL-6: Interleukin 6; IRE1a: Inositol-Requiring Enzyme 1 alpha; *JUN*: Jun proto-oncogene; *JUNB*: Jun B proto-oncogene; *Klkb1*: Kallikrein B1; *LIG4*: Ligase IV, DNA, ATP-dependent; MAPK: Mitogen-Activated Protein Kinase; *MCL1*: Myeloid cell leukemia sequence 1 (BCL2-related); *MDM2*: Human homologue of mouse double minute 2; MK167: Marker of Ki-67; *MRE11A*: MRE11 meiotic recombination 11 homolog A (*S. cerevisiae*); *MSH3*: mutS homolog 3 (*E. coli*); *MYC*: v-myc avian myelocytomatosis viral oncogene homolog; NAC: N-Acetyl Cysteine; NAFLD: Non-Alcoholic Fatty Liver Disease; *NAT1*: N-acetyltransferase 1 (arylamine N-acetyltransferase); NAT2: N-Acetyltransferase 2; *NFKB1*: Nuclear factor of kappa light polypeptide gene enhancer in B-cells 1; *Nrf2*: Nuclear factor erythroid 2-related factor 2; *Nupr1*: Nuclear Protein 1; *P53*: Tumor protein p53; *PCNA*: Proliferating cell nuclear antigen; PLA2: Phospholipase A2; *POLB*: Polymerase (DNA directed), Beta; PPAR-α: Peroxisome Proliferator-Activated Receptor alpha; *PPARa*: Peroxisome Proliferator-Activated Receptor alpha; PPM1: Protein Phosphatase 1; PPP6C: Protein Phosphatase 6 Catalytic Subunit; PROC: Protein C; *RAD1*: RAD1 homolog (*S. pombe*); *RAD51*: RAD51 homolog (*S. cerevisiae*); *Rad51*: RAD51 Recombinase; *RPL6*: Ribosomal Protein L6; *SCD1*: Stearoyl-CoA Desaturase 1; *SREBF1*: Sterol Regulatory Element-Binding Transcription Factor 1; *SREPB1*: Sterol Regulatory Element Binding Protein 1; *SULT1A1*: Sulfotransferase family, cytosolic, 1A, phenol-preferring, member 1; *SULT1B1*: Sulfotransferase Family 1 B Member 1; *SULT1B1*: Sulfotransferase Family 1 B Member 1; *SULT1C2*: Sulfotransferase Family 1 C Member 2; *TFDP1*:Transcription factor Dp-1; *TGFB1*: Transforming Growth Factor Beta 1; *TGFB2* Transforming growth factor. beta 2; *Thbs1*: Thrombospondin 1; *Thpo*: Thrombopoietin; *TIMP1*: TIMP metallopeptidase inhibitor 1; *TIMP2*: Tissue Inhibitor of Metalloproteinase 2; *TNF*: Tumor necrosis fator; *TNFAIP3*: Tumor necrosis factor. alpha-induced protein 3; *TNFAIP8*: TNF Alpha Induced Protein 8; TNFRSF1A-associated via death domain; *TNFRSF10A*: Tumor necrosis factor receptor superfamily. member 10a; *TOP2α*: Topoisomerase II Alpha; TRADD: TNFRSF1A-associated via death domain; *TTR*: Transthyretin; *TXNRD1*: Thioredoxin reductase 1; *UGT1*: UDP glucuronosyltransferase 1 family. polypeptide A6; *UGT1A1*: UDP glucuronosyltransferase 1 family. polypeptide A1; *UGT2B7*: UDP Glucuronosyltransferase 2B7; UHRF1: Ubiquitin-like with PHD and ring finger domains 1; *XPC*: Xeroderma pigmentosum. complementation group C; *XRCC2*: X-ray repair complementing defective repair in Chinese hamster cells 2.

#### 3.1.2. Transcriptomic Studies on CYN-Mediated Kidney Toxicity

The kidney is the second most studied organ at the transcriptomic level in relation to CYN toxicity, with oxidative stress as the main focus of study (Table 2). For instance, Diez-Quijada et al. [48] reported that CYN significantly altered the expression of *GPX1*, *CAT* (Catalase), and *SOD1* (Superoxide Dismutase 1) in vitro, while in vivo studies showed an increase in *Gst* and *Gpx* expression [44,45,47]. Additionally, CYN has been shown to affect tubular transport processes. Moraes et al. [49] demonstrated that exposure to CYN alters the expression of genes related to endocytosis and ionic homeostasis in renal tubular cells. Specifically, it disrupted the expression of *megalin* and *dab2* (DAB Adaptor Protein 2), which are both involved in receptor-mediated endocytosis, while *cubilin*, another gene involucrate in this process, remained unaffected. Furthermore, Na^+^/K^+^-ATPase (*ATP1A1*, ATPase Na^+^/K^+^ transporting subunit alpha 1), a key enzyme in ion transport and cellular homeostasis, was also altered. In addition, Diez-Quijada et al. [48] reported significant changes in genes related to xenobiotic metabolism (*CYP1A1*, *CYP1A2)*, DNA damage response (*TP53*, *CDKN1A*), and apoptosis regulation, with a significant alteration in *BCL2* (B-cell CLL/lymphoma 2) expression while *BAX* (Bcl2-Associated X protein) remained unchanged. Overall, transcriptomic studies indicate that CYN toxicity in the kidney occurs through the induction of oxidative stress and disruption of tubular transport and xenobiotic metabolism pathways.

#### 3.1.3. Transcriptomic Studies on CYN-Mediated Intestinal Toxicity

Despite oral exposure being the primary route for human contact with CYN, transcriptomic data concerning its adverse effects in the intestine remain limited (Table 3). Among the in vitro models, the human intestinal carcinoma cell line Caco-2 is one of the most widely used permanent cell lines in toxicological research, after rodent hepatocytes [13].

Bain et al. [27] investigated the effects of CYN in Caco-2 cells with a specific focus on determining the expression of the *CDKN1A* gene. They found that exposure to 1 µg/mL CYN for 24 h did not increase the expression of this gene. In other work, Huguet et al. [50] assessed the transcriptomic profile of Caco-2 cells following exposure to 1.6 µM CYN for 24 h. Their analysis identified 572 differentially expressed genes compared to control, with 522 genes being up-regulated and 50 down-regulated. The study reported that CYN induced the overexpression of proteins involved in transcriptional and post-transcriptional events, DNA damage repair, and modifications of nucleosomal histones. Regarding down-regulated genes, only three biological processes (response to calcium ion, metal ion, and inorganic ion) and one cellular component (membrane-bounded vesicle) were identified. These results highlighted new cell processes altered by CYN. Taken together, transcriptomic evidence on intestinal models remains scarce, but available data suggests that CYN can alter key cellular processes in intestinal cells, including transcriptional regulation, DNA damage, or chromatin remodeling, which may in turn affect intestinal barrier integrity.

**Table 2:** Transcriptomic analysis of CYN toxicity: Kidney.

Experimental Model	Exposure Concentration/Doses	Exposure Time	Route	Omics Type	Omics Technique	Pathway and Genes Analyzed	Reference
LLC-PK1 cells	0.1, 0.5, 1.0 µg/mL	12 h	In vitro	Transcriptomics	qRT-PCR	Tubular transport and endocytosis: *Megalin*, *Dab2*, *Cubilin*, *Atp1a1*	[49]
HEK293 cells	0.5, 5 µg/mL	4 h, 24 h	In vitro	Transcriptomics	qRT-PCR	Metabolism: *CYP1A1*, *CYP1A2* DNA damage: *TP53*, *CDKN1A* Oxidative stress: *CAT*, *GPX1*, *SOD1* Apoptosis/survival: *BCL2*, *BAX*	[48]
Tilapia (*Oreochromis niloticus*)	200 and 400 µg/kg bw	24 h	Gavage	Transcriptomics	RT-PCR	Oxidative stress: *Gpx*, *Gst*	[47]
Tilapia (*Oreochromis niloticus*)	Pure and liophilized cells 200 µg/kg + NAC	24 h	Oral	Transcriptomics	RT-PCR	Oxidative stress: *Gst*	[44]
Tilapia (*Oreochromis niloticus*)	200 µg/kg bw	24 h, 5 d	Gavage	Transcriptomics	RT-PCR	Oxidative stress: *Gpx*, *Gst*	[45]
			i.p. injection				

Abbreviations: *ATP1A1*: ATPase Na^+^/K^+^ transporting subunit alpha 1; *BAX*: Bcl2-Associated X protein; *BCL2*: B-cell CLL/lymphoma 2; *CAT*: catalase; *CDKN1A*: cyclin dependent kinase inhibitor 1A; *CYP1A1*: cytochrome P450 family 1 subfamily A member 1; *CYP1A2*: cytochrome P450 family 1 subfamily A member 2; Dab2: DAB Adaptor Protein 2; *GPX1/GPx*: glutathione peroxidase 1; *SOD1*: superoxide dismutase 1; *GST*: glutathione-S-transferase; *TP53*: tumor protein p53.

#### 3.1.4. Transcriptomic Studies on CYN-Mediated Immunotoxicity

Although investigations into the immunotoxicity of CYN remain scarce, it has been demonstrated that this cyanotoxin can elicit immunotoxic effects [8]. Immunotoxic effects of CYN evaluated through transcriptomics are shown in Table 3. Žegura et al. [51] investigated the effects of CYN on gene expression in human peripheral blood lymphocytes (HPBLs) following exposure to 0.5 µg/mL for 4 and 24 h. Their study evaluated the expression of genes involved in metabolism (*CYP1A1*, *CYP1A2*), DNA damage response (*P53*, *MDM2* (human homologue of mouse double minute 2), *GADD45α*, and *CDKN1A*), oxidative stress (*GPX1*, *SOD1*, *GSR* (glutathione-disulfide reductase gene), *GCLC* (glutamate-cysteine ligase catalytic subunit), and *CAT*), and apoptosis (*BCL-2*, *BAX*). CYN exposure resulted in the up-regulation of metabolism-related genes and affected the expression of genes involved in DNA damage response, apoptosis, and oxidative stress, with the exception of *CDKN1A* and *CAT*, which showed no alterations in their expression. More recently, Casas-Rodriguez et al. [52] exposed THP-1 and Jurkat cells to CYN at concentrations of 2.56 µM and 2.14 µM, respectively, for 4 and 24 h. After 24 h of exposure, both cell lines showed a pronounced pro-inflammatory gene expression profile. Specifically, in THP-1 cells, CYN up-regulated *IL-2* (Interleukin 2), *IL-6* (Interleukin 6), and *IFN-γ* (Interferon gamma), while in Jurkat cells, increased expression of *IL-2*, *TNF-α* (Tumor necrosis factor), and *IFN-γ* was observed. These results indicated that CYN could exert significant immunomodulatory effects on human immune cells.

In vivo experimental models used to study the immunotoxic effects of CYN include freshwater fish species, such as common carp (*Cyprinus carpio* L.), [53] and rodents, such as Sprague Dawley rats [54]. Sierolawska et al. [53] investigated the impact of CYN on the innate immune response in common carp by exposing phagocytic cells isolated from the head kidney to CYN concentrations up to 1 µg/mL for 24 h. They evaluated alterations in gene expression of pro-inflammatory (*IL-1β* (Interleukin 1 Beta), *TNF-α*) and anti-inflammatory (*IL-10* (Interleukin 10), *TGF-β* (Transforming Growth Factor Beta 1)) cytokines. After 24 h of exposure, no significant changes in *IL-10* gene expression were observed. However, a concentration-dependent up-regulation of *IL-1β* and *TNF-α* was detected. Interestingly, *TGF-β* gene expression showed its greatest increase with the lowest CYN concentration, whereas higher concentrations did not produce a similar increase. These findings suggest that CYN modulates the innate immune response in *Cyprinus carpio*, tilting it toward a predominantly pro-inflammatory profile. In other work, Diez-Quijada et al. [54] investigated the immunotoxic effects of CYN by analyzing mRNA expression levels of selected interleukin genes (*IL-1β*, *IL-2*, *IL-6*, *TNF-α*, and *IFN-γ*) in the thymus and spleen of male and female Sprague Dawley rats exposed orally to CYN at doses of 18.75, 37.5, and 75 µg/kg bw for 28 days. Their findings indicated that CYN primarily induced immunomodulation within the thymus, particularly at the highest dose evaluated (75 µg/kg). Specifically, in male rats, the expression of *IL-1β*, *IL-6*, *TNF-α*, and *IFN-γ* was altered, whereas in females, changes were observed in the expression of *IL-2*, *IL-6*, and *IFN-γ*. Conversely, the spleen exhibited a more restricted pattern of altered gene expression following CYN exposure, with modifications noted only for *IL-1β* and *IL-2* in males and for *TNF-α* and *IFN-γ* in females. These results highlight CYN’s capacity to modulate the immune response by altering the mRNA expression of several key interleukins in a tissue- and sex-specific manner.

#### 3.1.5. Transcriptomic Studies on CYN-Mediated Neurotoxicity

While specific studies on the neurotoxic effects of CYN are limited, its neurotoxicity has been previously documented [55]. To date, only two studies have used transcriptomic techniques to assess the neurotoxicity of CYN [56,57] (Table 3). Reid et al. [56] exposed undifferentiated mouse embryonic stem (mES) cells and embryoid bodies (EBs) to 1 µg/L CYN and assessed the impact of this cyanotoxin on *Oct4*, *Brachyury*, and *Nestin*, three genetic markers crucial for characterizing gene expression patterns during differentiation. Their findings indicated that CYN did not affect the expression of these genes, suggesting that, at this concentration, CYN does not influence genes involved in embryonic and neural development. In a recent study, Hinojosa et al. [57] performed a transcriptomic analysis on SH-SY5Y neuroblastoma cells following a 6-day differentiation period and exposure to 0.097 µM of CYN. Out of 93 differentially expressed genes, 5 were significantly reduced: *NTNG2* (Netrin G2), *KCNJ11* (Potassium Inwardly Rectifying Channel Subfamily J Member 11), *SLC18A3* (Solute Carrier Family 18 Member A3), *APOE* (Apolipoprotein E), and *SEMA6B* (Semaphorin 6B), all of which are involved in neurodevelopment processes. Although information on CYN neurotoxicity is scarce, transcriptomic findings suggest a potential effect of this cyanotoxin on neurodevelopment and provide valuable insights into the mechanisms underlying CYN-induced neurotoxicity.

**Figure 1:**
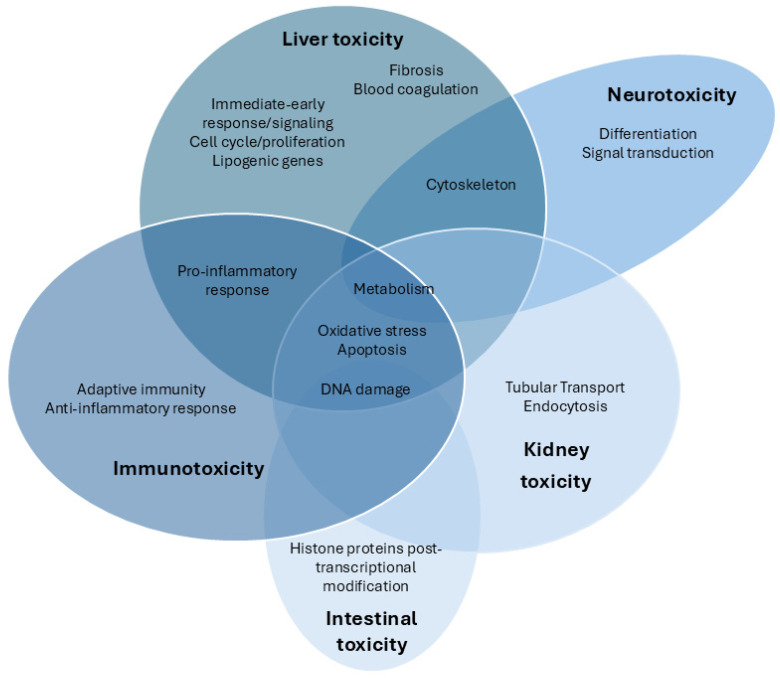
Venn diagram of the transcriptomic pathways analyzed after exposure to CYN in different target organs and systems.

We comprehensively summarized the current studies on the toxic effects of CYN based on transcriptomic analysis. As shown in Figure 1, the available transcriptomic data on CYN toxicity primarily focus on the hepatic, immune system, and kidney, followed by the intestinal and neuronal levels. Other studies also involve various in vitro models, which will be explained below.

**Table 3:** Transcriptomic analysis of CYN toxicity in other in vitro and in vivo models.

Experimental Model	Exposure Concentration/Doses	Exposure Time	Route	Omics Type	Omics Technique	Pathway and Genes Analyzed	Reference
				**In vitro**			
				**Intestinal models**			
Caco-2 cells	1 µg/mL	24 h	In vitro	Transcriptomics	qRT-PCR	DNA damage: *CDKN1A*	[27]
Differentiated Caco-2 cells	1.6 µM	24 h	In vitro	Transcriptomics	RT-qPCR	Transcription, post-transcriptional modifications of RNA, and histone proteins post-transcriptional modification: *POLR2D*, *POLR2L*, *MED6*, *DDX20*, *KAT5*, *MYST1*, *EHMT2*, *RPLP0*	[50]
				**Immune models**			
HPBLs	0.5 µg/mL	4 h 24 h	In vitro	Transcriptomics	qRT-PCR	Metabolism: *CYP1A1* and *CYP1A2* DNA damage responsive genes: *P53*, *MDM2*, *GADD45α* and *CDKN1A* Oxidative stress: *GPX1*, *SOD1*, *GSR*, *GCLC* and *CAT* Apoptosis/survival: *BCL-2* and *BAX*	[51]
Jurkat cells	2.14 µM	4 h 24 h	In vitro	Transcriptomics	RT-qPCR	Adaptive immunity: *IL-2* Pro-inflammatory: *IL-6*, *IL-8*, *TNF-α*, *INF-γ*	[52]
THP-1 cells	2.56 µM	4 h 24 h	In vitro	Transcriptomics	RT-qPCR	Adaptive immunity: *IL-2* Pro-inflammatory: *IL-6*, *IL-8*, *TNF-α*, *INF-γ*	[52]
				**Neural models**			
Undifferentiated D3 mES cells and differentiated EBs	1 µg/L	Up to 9 days	In vitro	Transcriptomics	RT-PCR	Differentiation: *Oct4*, *Brachyury*, *Nestin*	[56]
SH-SY5Y cells	0.097 µM	6 days	In vitro	Transcriptomics	RT-qPCR	93 selected genes. Signal Transduction; Cell Adhesion/Extracellular Matrix; Cytoskeleton; Neurotransmission; Cell Signaling; Protein Metabolism. *ACTA2*, *ADCY5*, *AGRN*, *APLNR*, *APOE*, *BDNF*, *BHLHE40*, *BMP7*, *CACNA1E*, *CACNA1G*, *CACNA2D2*, *CDH23*, *CHRM1*, *CHRM2*, *CHRM3*, *CHRNA7*, *CNR1*, *CREB1*, *CRHR1*, *CSPG5*, *CYP26A1*, *DPYSL3*, *EDNRA*, *EFNB2*, *EGFR*, *EIF4EBP1*, *ERBB3*, *FGF1*, *FGFR4*, *GABRD*, *GABRG3*, *GABRP*, *GAL*, *GAP43*, *GCH1*, *GDF15*, *GFRA1*, *GFRA2*, *GNG7*, *GRIN2C*, *GRIN2D*, *GRM1*, *GRM7*, *GSN*, *HSP90AB1*, *ITGA1*, *ITGA3*, *ITGB4*, *KCNJ11*, *KCNN3*, *KCNQ2*, *MAPT*, *MYC*, *NFKB2*, *NLGN1*, *NLGN3*, *NR3C1*, *NRXN1*, *NTNG2*, *NTRK1*, *NTRK2*, *OPRD1*, *PAK6*, *PARK2*, *PDE4A*, *PDGFRA*, *PDLIM7*, *PGF*, *PIK3CD*, *PRKCG*, *RAPGEF4*, *RASD2* *RASGRP2*, *RET*, *RGS9*, *RHOQ*, *RND2*, *RPLP1*, *RYR1*, *RYR2*, *S1PR3*, *SEMA3F*, *SEMA4A*, *SEMA5A*, *SEMA6B*, *SH2D3C*, *SLC18A3*, *SLIT1*, *SNCA*, *SNCAIP*, *SRGAP3*, *TGFB1*, *TP53*, *TUBA4A* *VCAN*	[57]
				**Dermal models**			
HDFs	1, 2.5, or 5 µg/mL 1 µg/mL	6 h 24 h	In vitro	Transcriptomics	qRT-PCR	DNA damage: *CDKN1A*, *GADD45α*, *MDM2* Apoptosis/survival: *BAX*	[27]
				**Endothelial model**			
HUVECs	2, 20, 200, and 2000 nM	24 h	In vitro	Transcriptomics	RT-PCR	Cytoskeleton: *ITGB1*, *RHO*, *ROCK*, *MLC-1*, *VIM-1* Apoptosis/survival: *BAX*, *BCL-2*	[58]
				In vivo			
				**Immune models**			
Phagocytic cells from common carp (*Cyprinus carpio* L.)	0.05, 0.1, 0.5 or 1 µg/ml	24 h	In vivo	Transcriptomics	RT-PCR	Pro-inflammatory: *Il-1β*, *Tnf-α* Anti-inflammatory: *Il-10*, *Tgf-β*	[53]
Sprague Dawley rats (thymus, spleen)	18.75, 37.5 and 75 µg/kg bw/day	28 days	In vivo oral	Transcriptomics	RT-qPCR	Pro-inflammatory: *Il-1β*, *Il-6*, *Tnfα*, *Inf-γ* Anti-inflammatory: *Il-2*	[54]

Abbreviations: 2DE: Two-dimensional electrophoresis; *ACTA2*: Actin Alpha 2; *ADCY5*: Adenylate Cyclase 5; *AGRN*: Agrin; *APLNR*: Apelin Receptor; *APOE*: Apolipoprotein E; bw: body weight; *BAX*: BCL2 associated X, apoptosis regulator; *BCL-2*: B-cell leukemia/lymphoma 2 protein; *BDNF*: Brain Derived Neurotrophic Factor; *BHLHE40*: Basic Helix-Loop-Helix Family Member E40; *BMP7*: Bone Morphogenetic Protein 7; *CACNA1E*: Calcium Voltage-Gated Channel Subunit Alpha1 E; *CACNA1G*: Calcium Voltage-Gated Channel Subunit Alpha1 G; *CACNA2D2*: Calcium Voltage-Gated Channel Auxiliary Subunit Alpha2delta 2; Caco-2: Human colorectal adenocarcinoma cell line; *CAT*: Catalase; *CDH23*: Cadherin Related 23; *CDKN1A*: Cyclin-Dependent Kinase Inhibitor 1A; *CHRM1*: Cholinergic Receptor Muscarinic 1; *CHRM2*: Cholinergic Receptor Muscarinic 2; *CHRM3*: Cholinergic Receptor Muscarinic 3; *CHRNA7*: Cholinergic Receptor Nicotinic Alpha 7 Subunit; *CNR1*: Cannaboid Receptor 1; *CREB1*: cAMP Responsive Element Binding Protein 1; *CRHR1*: Corticotropin Releasing Hormone Receptor 1; *CSPG5*: Chondroitin Sulfate Proteoglycan 5; *CYP1A1*: cytochrome P450, family 1, subfamily A, polypeptide 1; *CYP1A2*: cytochrome P450, family 1, subfamily A, polypeptide 2; *CYP26A1*: Cytochrome P450 Family 26 Subfamily A Member 1; *DPYSL3*: Dihydropyrimidinase Like 3; EB: Embroid bodies; *EDNRA*: Endothelin Receptor Type A; *EFNB2*: Ephrin B2; *EGFR*: Epidermal Growth Factor Receptor; *EIF4EBP1*: Eukaryotic Translation Initiation Factor 4E Binding Protein 1; *ERBB3*: Erb-B2 Receptor Tyrosine Kinase 3; *FGF1*: Fibroblast Growth Factor 1; *FGFR4*: Fibroblast Growth Factor Receptor 4; *GABRD*: Gamma-Aminobutyric Acid Type A Receptor Subunit Delta; *GABRG3*: Gamma-Aminobutyric Acid Type A Receptor Subunit Gamma3; *GABRP*: Gamma-Aminobutyric Acid Type A Receptor Subunit Pi; *GADD45α*: Growth arrest and DNA-damage-inducible, Alpha; *GAL*: Galanin; *GAP43*: Growth Associated Protein 43; *GCH1*: GTP Cyclohydrolase 1; GD: gestational days; *GDF15*: Growth Differentiation Factor 15; *GCLC*: glutamate-cysteine ligase catalytic subunit; *GFRA1*: GDNF Family Receptor Alpha 1; *GFRA2*: GDNF Family Receptor Alpha 2; *GNG7*: G Protein Subunit Gamma 7; *GPX1*: Glutathione peroxidase 1; *GRIN2C*: Glutamate Ionotropic Receptor NMDA Type Subunit 2C; *GRIN2D*: Glutamate Ionotropic Receptor NMDA Type Subunit 2D; *GRM1*: Glutamate Metabotropic Receptor 1; *GRM7*: Glutamate Metabotropic Receptor 7; *GSN*: Gelsolin; *GSR*: glutathione-disulfide reductase gene; HDFs: Human dermal fibroblasts; *HSP90AB1*: Heat Shock Protein 90 Alpha Family Class B Member 1; HPBLs: Human peripheral blood lymphocytes; HUVECs: Human umbilical vein endothelial; i.p.: intraperitoneal; IL-1β: interleukin-1β; IL-2:Interleukin 2; IL-6: Interleukin 6; IL-8: Interleukin 8; IL-10: interleukin-10; INF-γ: interferon-gamma; *ITGA1*: Integrin Subunit Alpha 1; *ITGA3*: Integrin Subunit Alpha 3; *ITGB1*: Integrin beta-1 protein; *ITGB4*: Integrin Subunit Beta 4; Jurkat: cell line derived from the peripjeral blood of a patient with aceute T-cell leukemia; *KCNJ11*: Potassium Inwardly Rectifying Channel Subfamily J Member 11; *KCNN3*: Potassium Calcium-Activated Channel Subfamily N Member 3; *KCNQ2*: Potassium Voltage-Gated Channel Subfamily Q Member 2; *MAPT*: Microtubule Associated Protein Tau; *MDM2*: Human homologue of mouse double minute 2; mES: mouse embryonic stem; MLC-1: megalencephalic leukoencephalopathy with subcortical cysts 1; *MYC*: MYC Proto-Oncogene, BHLH Transcription Factor; *NFKB2*: Nuclear Factor Kappa B Subunit 2; *NLGN1*: Neuroligin 1; *NLGN3*: Neuroligin 3; *NR3C1*: Nuclear Receptor Subfamily 3 Group C Member 1; *NRXN1*: Neurexin 1; *NTNG2*: Netrin G2; *NTRK1*: Neurotrophic Receptor Tyrosine Kinase 1; *NTRK2*: Neurotrophic Receptor Tyrosine Kinase 2; *OPRD1*: Opioid Receptor Delta 1; *P53*: P53: Tumor protein p53; *PAK6*: P21 (RAC1) Activated Kinase 6; *PARK2*: Parkin RBR E3 Ubiquitin Protein Ligase; *PDE4A*: Phosphodiesterase 4A; *PDGFRA*: Platelet Derived Growth Factor Receptor Alpha; *PDLIM7*: PDZ And LIM Domain 7; *PGF*: Placental Growth Factor; *PIK3CD*: Phosphatidylinositol-4,5-Bisphosphate 3-Kinase Catalytic Subunit Delta; *PRKCG*: Protein Kinase C Gamma; *RAPGEF4*: Rap Guanine Nucleotide Exchange Factor 4; *RASD2*: RASD Family Member 2; *RASGRP2*: RAS Guanyl Releasing Protein 2; *RET*: Ret Proto-Oncogene; *RGS9*: Regulator Of G Protein Signaling 9; Rho: rhodopsin; *RHOQ*: Ras Homolog Family Member Q; *RND2*: Rho Family GTPase 2; ROCK: Rho-associated coiled-coil containing protein kinase 1; *RPLP1*: Ribosomal Protein Lateral Stalk Subunit P1; RT-qPCR: Quantitative reverse transcription polymerase chain reaction; *RYR1*: Ryanodine Receptor 1; *RYR2*: Ryanodine Receptor 2; *S1PR3*: Sphingosine-1-Phosphate Receptor 3; *SEMA3F*: Semaphorin 3F; *SEMA4A*: Semaphorin 4A; *SEMA5A*: Semaphorin 5A; *SEMA6B*: Semaphorin 6B; *SH2D3C*: SH2 Domain Containing 3C; SH-SY5Y: human neuroblastoma cell line; *SLC18A3*: Solute Carrier Family 18 Member A3; *SLIT1*: Slit Guidance Ligand 1; *SNCA*: Synuclein Alpha; *SNCAIP*: Synuclein Alpha Interacting Protein; *SOD1*: Superoxide Dismutase; *SRGAP3*: SLIT-ROBO Rho: GTPase Activating Protein 3; TGFβ: transforming growth factor; *TGFB1*: Transforming Growth Factor Beta 1; THP-1: cell line derived from peripheral blood from a patient with acute monocytic leukemia; TNF-α: tumor necrosis faltor-alpha; *TP53*: Tumor Protein P53; *TUBA4A*: Tubulin Alpha 4a; *VCAN*: Versican; *VIM-1*: Vimentin.

#### 3.1.6. Transcriptomic Studies on CYN in Other In Vitro Models

Based on the current literature, only two studies have utilized transcriptomic techniques to evaluate CYN toxicity in dermal cells [27] and endothelial cells [58] (Table 3).

Bain et al. [27] investigated the impact of CYN on the gene expression of p53 target genes (*CDKN1A*, *GADD45α*, *BAX*, and *MDM2*) in human dermal fibroblasts (HDFs). Cells were exposed to different concentrations of CYN (up to 5 µg/mL for 6 h and 1 µg/mL for 24 h). After 6 h of exposure, a significant increase in the expression of all analyzed genes was observed, showing a positive correlation with CYN concentration. At 24 h, gene expression levels remained elevated compared to the control. These results indicated early activation of the p53 pathway in response to CYN exposure.

Research into the vascular toxicity of CYN is limited, despite its known toxic effects. Wang et al. [58] investigated CYN toxicity in human umbilical vein endothelial cells (HUVECs) after 24 h of exposure to concentrations up to 2000 nM. Their analysis focused on the expression of key genes associated with the Rho/ROCK signaling pathway, cytoskeleton, and apoptosis, including *ITGB1* (Integrin β-1), *Rho* (Rhodopsin), *ROCK* (Rho-associated coiled-coil containing protein kinase 1), *MLC-1* (Megalencephalic leukoencephalopathy with subcortical cysts 1), *VIM-1* (Vimentin), *Bax*, and *Bcl-2*. A significant, concentration-dependent down-regulation of *ITGB1* was reported. Conversely, *Rho* and *ROCK* expression were only affected at the highest CYN concentrations (200 and 2000 nM). *MLC-1* showed up-regulation at lower CYN concentrations (2 and 20 nM), while *VIM-1* expression notably decreased in a concentration-dependent manner. Regarding apoptotic gene expression, *Bax* levels increased with rising CYN concentrations, whereas *Bcl-2* expression was altered only at the highest concentration. These findings collectively suggest that CYN could induce apoptosis through its influence on the Rho/ROCK signaling pathway.

#### 3.1.7. Transcriptomic Studies with CYN in Other In Vivo Models

Transcriptomic studies have also been conducted in the freshwater cladoceran *Daphnia magna*, providing insights into the molecular mechanisms of CYN toxicity in invertebrates. He et al. [59] showed that CYN alters gene expression related to oxidative stress (through the repression of *Hem* genes involved in protoheme synthesis, which is crucial for antioxidant defense), proteolysis, energy metabolism, lipid metabolism, and neurological function. These alterations suggest compromised antioxidant defenses, impaired ATP production, increased protein degradation, and potential reproductive and neurological effects. In a subsequent study, He et al. [60] compared the effects of pure CYN with those of an algal extract, revealing that despite similar phenotypic toxicity, the molecular mechanisms differ. Interestingly, while both compounds induce oxidative stress, only the algal extract activated antioxidant-related genes, whereas pure CYN failed to trigger oxidative defense pathways. These findings suggest that the algal extract may cause more effective compensatory cellular responses to oxidative damage than CYN alone.

Transcriptomic analysis was also used to investigate the effects of CYN on the green alga *Scenedesmus bijugatus* [61]. The authors reported that CYN activated genes involved in the PI3K/Akt-cGMP/PKG signaling pathway (*prkg1* (Protein kinase cGMP-dependent 1), *gucy1A1* (Guanylate cyclase 1 soluble subunit alpha 1), *akt* (serine/threonine-protein kinase)), which may contribute to nitrogen allocation and support alkaline phosphatase (ALP) production. Specifically, they observed an up-regulation of genes related to leucine biosynthesis (*leuB* (3-isopropylmalate dehydrogenase) and *sds* (serine dehydratase)), which is one of the most abundant amino acids in ALP, ALP enzyme encoding (phoD, alkaline phosphatase), and protein export (sec1β, SEC61 translocon subunit beta). These transcriptomic changes suggest that CYN acts as an infochemical signal that, when perceived by *S. bijugatus*, stimulates the production and secretion of ALP. This enhances the regeneration of inorganic phosphorus and favors the growth of the CYN producer, *Cylindrospermopsis raciborskii*, in phosphorus-limited environments.

Finally, some studies have shown that CYN induces transcriptomic alterations in *Danio rerio*, affecting development and reproduction. In embryos, CYN increased the expression of genes involved in oxidative stress (*SOD1*, *CAT*, and *GPX1*) and apoptosis (*p53*, *Bax*), and decreased the expression of *Bcl-2*, which may underlie developmental defects [62]. In testes, CYN altered gonadotropin-responsive genes such as *fshr* (Follicle-stimulating hormone receptor), *lhr* (Luteinizing hormone/choriogonadotropin receptor), and *igf3* (Insulin-like growth factor 3) under human chorionic gonadotropin (hCG) stimulation. Although *srd5a2* (steroid-5-alpha-reductase, alpha polypeptide 2a) and *3βhsd* (hydroxy-delta-5-steroid dehydrogenase, 3 beta- and steroid delta-isomerase 2), which are involved in androgen biosynthesis and sperm function, remained unchanged, the results pointed to a disruption of spermatogenesis regulation [63]. More recently, Li et al. [64] found widespread gene expression changes in gonads after exposure to *Oscillatoria* sp. (CYN producer), with stronger effects in males. Alterations of steroidogenic (*cyp19a1a* (cytochrome p450 family 19 subfamily A polypeptide 1a), *cyp11a2* (cytochrome p450 family 11 subfamily A member 2), *hsd17b1* (hydroxysteroid (17-beta) dehydrogenase 1), and *hsd17b3* (hydroxysteroid (17-beta) dehydrogenase 3)), metabolic (*aldob* (aldolase), *pgm2* (phosphoglucomutase 2), *g6pca.2* (glucose-6-phosphatase catalytic subunit 1a, tandem duplicate 2)), and extracellular matrix-related (*lamb1a* (laminin beta 1a), *col4a5* (collagen type IV alpha 5 chain), and *itga3b* (integrin alpha 3b)) genes, along with pathway disruptions (PPAR signaling, ferroptosis, or cell adhesion), suggest that chronic CYN exposure affects reproductive and growth regulation in zebrafish.

### 3.2. Proteomics

#### 3.2.1. Proteomic Studies on CYN-Mediated Liver Toxicity

In recent years, a limited yet growing number of studies have employed a proteomic approach to investigate the effects of CYN. In this regard, as in transcriptomic analyses, human liver cells have been the most frequently used experimental model (Table 4). Proteomic data derived from these studies have revealed alterations in key cellular pathways, including oxidative stress, apoptosis, protein folding, and lipid metabolism, among others [27,34,36,39,65]. With respect to oxidative stress, analyses of hepatocyte models revealed an up-regulation of protective mechanisms following CYN exposure, including increased expression of GPx and multidrug resistance protein 3 (MRP3) in HepG2 [65], as well as a slight elevation in *SOD1* and *CAT* levels in both HepG2 and SK-Hep1 cells [34]. In in vivo models, alterations in antioxidant system proteins have also been reported in the liver. Specifically, an increase in soluble *GST* levels was observed in the liver of tilapia following exposure to pure CYN for 24 h, after both oral and i.p. administration [45,47].

As for protein folding, the studies showed different responses. Liebel et al. [65] observed a dual effect; while certain chaperone levels, such as HSP70 (Heat shock protein 70 kDa isoform 8 variant 2) and GRP75 (75 kDa glucose regulated protein), decreased, which could be related to early cytotoxicity, others, including HSC71 (Heat shock cognate 71 kDa isoform 1), HSP90 (Heat shock protein 90α isoform 1), GRP78 (78 kDa glucose-regulated protein), HSP60 (Mitochondrial heat shock 60 kDa protein 1 variant 1), and protein disulfide isomerase precursor (ER) increased, evidencing activation of the UPR. In contrast, Niture et al. [34] observed no significant changes in UPR markers. These authors further examined the impact of CYN on lipid metabolism and hepatic steatosis. Their findings showed elevated expression of acetyl-CoA carboxylase (ACC) in HepG2, and both ACC and stearoyl-CoA desaturase-1 (SCD1) in SK-Hep1, while levels of SREBP-1 (Sterol regulatory element-binding protein-1), FABP1 (Fatty acid-binding protein-1), FASN (Fatty Acid Synthase), and PPARα (Peroxisome Proliferator-Activated Receptor alpha) remained unchanged. In parallel, CYN partially activated the AKT/mTOR pathway and slightly increased p62 expression, suggesting that impaired autophagy may contribute to lipid accumulation [34]. Complementing these studies, Chowdhury et al. [39] detected a significant increase in perilipin 2 (ADRP) peptides in three-dimensional HepG2 spheroids. Taken together, these studies demonstrate that CYN can promote hepatic steatosis.

With respect to proteins involved in apoptotic mechanisms, Bain et al. [27] reported increased levels of the tumor suppressor p53 in C3A cells (a HepG2-derived line) following 48 h of CYN exposure, suggesting activation of the p53-mediated apoptotic pathway. In addition, Vanova et al. [36] found pronounced PARP cleavage and caspase-3 activation in progenitors (day 10) and immature cells (day 15) and CCTL4 cells, whereas mature hepatocytes (day 20) showed an increase in necrotic markers. Conversely, Niture et al. [34], using lower concentrations of CYN (10–250 nM) and longer exposure durations (72 h), observed no significant changes in cleaved-PARP or caspase-3 expression in either HepG2 or SK-Hep1 cells. Collectively, these findings indicate that CYN can activate both p53-dependent and caspase-mediated apoptotic pathways, but the outcome depends on toxin concentration, exposure duration, intrinsic p53 levels, and the differentiation state of the target cells.

Other alterations identified through proteomic analysis in liver cell models exposed to CYN included disruptions in energy metabolism, as indicated by a decrease in glutathione-insulin transhydrogenase and an up-regulation of several proteins involved in glycolysis (fructose 1,6-bisphosphate aldolase, glyceraldehyde-3-phosphate dehydrogenase, and lactate dehydrogenase). Moreover, changes in cytoskeletal integrity were evident through reduced expression of α-actin and β-actin proteins [65]. On the other hand, Raska et al. [38] demonstrated that non-cytotoxic concentrations of CYN (0.1–1 µM) in HL1-hT1 human liver stem cells triggered time-dependent activation of mitogen-activated protein kinase (MAPK) signaling, evidenced by a marked rise in extracellular signal-regulated kinase 1/2 (ERK1/2) phosphorylation and p38 activation. Overall, these findings reinforce the effects of CYN observed at the transcriptional level in the liver and indicate that CYN triggers oxidative stress and apoptosis, and disrupts protein folding in this organ.

#### 3.2.2. Proteomic Studies on CYN-Mediated Kidney Toxicity

Given the nephrotoxic potential of CYN, Diez-Quijada et al. [48] examined the proteomic response of HEK293 cells to CYN alone and in combination with MC-LR, revealing alterations in proteins involved in diverse processes, including RNA and protein transport, Golgi structure and intracellular transport, immunity, lipid metabolism, protein ubiquitination and degradation, cell adhesion, gene transcription and translation, and protein biosynthesis. In in vivo experiments in tilapia, Puerto et al. [47] reported a dose-dependent increase in GST abundance in the kidney after gavage administration of 200 µg/kg bw CYN. Extending this work, Gutiérrez-Praena et al. [45] examined renal GST levels in tilapia as a function of administration route (oral or i.p.) and the time of sacrifice (24 h vs. 5 days). They found that only fish exposed i.p. and sacrificed after 5 days exhibited a significant increase in *Gst* abundance (Table 4). These proteomic findings indicate that CYN can disrupt key renal processes related to protein metabolism and detoxification.

#### 3.2.3. Proteomic Studies on CYN-Mediated Intestinal Toxicity

To our knowledge, only one study has employed proteomic analysis to assess the effects of CYN at the intestinal level (Table 4). Specifically, in differentiated Caco-2 cells exposed to a sub-toxic concentration of CYN, elevated levels of the histone modifiers KAT5 (Lysine acetyltransferase 5), MYST1 (Histone acetyltransferase 1), and EHMT2 (Euchromatic histone lysine methyltransferase 2) were observed, along with an increase in acetylation of histone H2A at lysine 5 and dimethylation of histone H3 at lysine 4. These findings suggest that CYN activates DNA repair and transcription mechanisms, probably as a response to DNA damage [50].

#### 3.2.4. Proteomic Studies on CYN-Mediated Bronchial Epithelial Cell Toxicity

The effects of CYN have also been investigated in 16HBE14o-human airway epithelial cells (Table 4). This study identified up to 5000 proteins, of which 98 showed CYN-mediated alterations in abundance. Altered proteins were grouped into pathways such as protein stability (e.g., down-regulation of cystatin-C and SPINT2 (Serine peptidase inhibitor, Kunitz type 2)), extracellular matrix and cell adhesion (in particular SPARC (Secreted protein acidic and rich in cysteine), agrin (APLNR), laminin α-5 and claudin-6) and cell division (including FAM83D (Family with sequence similarity 83 member D), SAPCD2 (Suppressor anaphase-promoting complex domain 2), and CEP55 (Centrosomal protein of 55 kDa)). These results suggest these physiological cell mechanisms as potential CYN targets in this experimental model [66].

#### 3.2.5. Proteomic Studies with CYN in Different Vivo Models

Proteomic investigations have also been carried out using other in vivo models (Table 4). In bivalves (*Mytilus galloprovincialis* and *Corbicula fluminea*) exposed to CYN-producing *C. raciborskii*, a decrease in HSP60 was observed that was associated with a stress response [67]. This study also revealed differential expression of structural proteins (tubulin and actin isoforms) and energy production proteins (ATP synthase β subunit and triosephosphate isomerase), indicating cytoskeletal disruption and cell injury. Furthermore, extrapallial fluid protein (EP), which is involved in metal transport and calcium fixation, was down-regulated only in the gills of *M. galloprovincialis* exposed to CYN-producing cyanobacteria, highlighting that CYN may cause additional physiological impairments [67]. Extending these findings, Oliveira et al. [68] showed that 15-day exposure of *M. galloprovincialis* to *Microcystis aeruginosa* (producer of MC-LR), *Chrysosporum ovalisporum* (producer of CYN), or their mixture changed the digestive-gland proteome, particularly under co-exposure conditions. Alterations were observed in multiple metabolic pathways, such as protein folding and stabilization, cytoskeleton organization, and gene transcription and translation, among others.

Although several studies have investigated the impact of CYN in mammals [18], to our knowledge, only one study has applied a proteomic approach to assess its toxicity. Moraes et al. [69] studied CYN-induced renal tubular damage in 10-week-old male BALB/c mice exposed to a single i.p. administration of purified CYN. Urinary protein profiling revealed pronounced proteinuria with elevated excretion of low-molecular-weight proteins, such as albumin, suggesting alteration of the proximal tubular reabsorption process. Similarly, only one study has assessed the effects of CYN on protein abundance in vegetables, focusing exclusively on lettuce (*Lactuca sativa*) [70]. After five days of exposure, 2-DE analysis revealed 68 protein spots with significantly altered abundance in the CYN treatment and 286 spots in the combined CYN + MC-LR treatment, indicating a synergistic interaction between the two cyanotoxins. Notably, in the CYN experiment, higher concentrations caused more pronounced changes, whereas in co-exposure, the greatest response occurred at the lowest concentration. In both treatments (CYN and CYN + MC-LR), the affected proteins were functionally classified into key pathways, including photosynthesis and carbon metabolism, ATP synthesis, stress/defense responses, and protein folding [67]. Overall, these proteomic studies across species suggest that CYN mainly affects proteins involved in metabolism, cytoskeletal organization, and stress response.

**Table 4:** Proteomic analysis of CYN toxicity.

Experimental Model	Exposure Concentration/Doses	Exposure Time	Route	Omics Type	Omics Technique	Pathway and Proteins Analyzed	Reference
**Hepatic models**							
HDFs, HepG2 and C3A cells	10–1000 ng/mL	48 h	In vitro	Proteomics	Inmunoblotting	DNA damage: P53 protein	[27]
Liver of tilapia (*Oreochromis niloticus*)	200 or 400 µg/kg bw	Single dose and sacrificed 24 h after administration	Gavage	Proteomics	Western blot	Oxidative stress: GST	[47]
Liver of tilapia (*Oreochromis niloticus*)	200 µg/kg bw	Sacrificed 24 h or 5 days after administration	Gavage or i.p. injection	Proteomics	Western blot	Oxidative stress: GST	[45]
HepG2 cells	10 µg/L	24 h	In vitro	Proteomics	2-DE gels and MALDI-TOF-TOF	Protein folding: HSP70, GRP75, HSC71, HSP90, protein disulfide isomerase-related 5, PDI isomerase precursor, GRP78, and HSP60. Antioxidant defense: GSHPx, MRP3 Energy metabolism, and biosynthesis: glutathione-insulin transhydrogenase, fructose 1,6-bisphosphate aldolase complexed with fructose 1,6-biphosphate, GADPHe, lactate dehydrogenase, and GRP78. Cell signaling and tumorigenic potential: protein kinase A catalytic subunit β, chain A, tapasin ERP57 heterodimer, GPCRs, and heterogeneous nuclear ribonucleoproteins A2/B1. Cytoskeleton structure: keratin, α-actin, β-actin, α-spectrina, α-tubulin, and β-5 tubulin.	[65]
hESCs CCTL14 at various stages of differentiation to hepatocytes	1 µM	24 or 48 h	In vitro	Proteomics	Western blotting	Apoptosis/survival: cleaved caspase 3, uncleaved procaspase 3, and cleaved PARP.	[36]
HL1-hT1	0.1 and 1 µM	Series of time points between 0.1 and 48 h	In vitro	Proteomics	SDS-PAGE and Western blotting	MAPK signal transduction pathways: ERK1/2, p38	[38]
HepG2 and SK-Hep1 cells	1 to 250 nM	72 h	In vitro	Proteomics	NuPAGE and Western blotting	Apoptosis/survival: PARP, pro-caspase-3, and cleaved caspase 3. Inflammatory signaling: IL-6, TNFAIP8. Oxidative stress: SOD1 and CAT. UPR: pERK, BIP, peIF2α, and ATF6. Lipogenic proteins: FASN, ACC, SCD1, FABP1, SREBP1, and PPARα. AKT/mTOR pathways: pS2448-mTOR, mTOR, pS473-AKT, and AKT. Cellular autophagy pathways: LC3B, beclin1, 4EBP1, p62. Fibrosis signaling: p21, TIMP2, MMP2, TGFβ.	[34]
HepG2 3D cell spheroids	1 µM	48 h	In vitro	Proteomics	LC-MS/MS	Lipid metabolism: PAT proteins analysis.	[39]
**Intestinal models**							
Differentiated Caco-2 cells	1.6 µM	24 h	In vitro	Proteomics	Immunolabelling	Chromatin remodeling: KAT5, MYST1, EHMT2, H2A, H4, and H3.	[50]
**Renal models**							
Kidney of tilapia (*Oreochromis niloticus*)	200 or 400 µg/kg bw	Single dose and sacrificed 24 h after administration	Gavage	Proteomics	Western blot	Oxidative stress: GST.	[47]
Kidney of tilapia (*Oreochromis niloticus*)	200 µg/kg bw	Single dose and sacrificed 24 h or 5 days after administration	Gavage or i.p. injection	Proteomics	Western blot	Oxidative stress: GST.	[45]
HEK293 cells	0.5 and 1 µg/mL CYN alone or mixed with 1 µg/mL MC-LR	24 h	In vitro	Proteomics	FASP, LC-MS/MS	Cellular metabolism: CBR1 and PGM2. Lipid metabolism: prosaposin and ACAA2. Cell adhesion: moesin, ITGB1, and FERMT2. Protein metabolism: BLMH. Protein regulation: RANBP2, STUB1, SUGT1, and CLPP. Protein synthesis: EIF2B1, TCOF1, RPS5, and LUC7L3. Protein transport: COPG1.	[48]
**Bronchial epithelial models**							
Immortalized human bronchial epithelial cells (16HBE14o-)	5 µmol/L CYN for SDS-PAGE and 1, 2.5 or 5 µmol/L for Western blotting	20 h for SDS-PAGE and between 0 and 36 h for Western blotting	In vitro	Proteomics	1 D SDS-PAGE, QExactive Plus and Western blotting	Protein stability: Cystatin-C and SPINT2. Cellular adhesion and integration in the extracellular. matrix: ITIH, SPARC, agrin, laminin subunit α-5, TIMP2, and claudin-6. Synthesis of membrane or secretory proteins: ribosome binding protein 1. Cell proliferation, cell cycle regulation and cytokinesis: FAM83D, SAPCD2, CEP55, p16Ink4a, p21Cip1, Arf, ASF1A, mitotic cyclin B1, G1/S-specific cyclin D1, cyclin D3, and RecQ-mediated genome instability protein 2.	[66]
**Other in vivo models**							
*Mytilus galloprovincialis* and *Corbicula fluminea*	5 × 10^5^ cells/mL CYN-producing *Cylindrospermopsis raciborskii* (equivalent to 0.072 µg CYN/L)	6 days	In vivo	Proteomics	2-DE gels and MALDI-TOF-TOF MS	Cytoskeleton structure: actin and tubulin isoforms. Oxidative stress: HSP60. Energy production: ATPase β subunit and triosephosphate isomerase. Calcium-binding and metal transport: EP.	[67]
Lettuce (*Lactuca sativa L.*)	1–100 µg/L CYN alone or mixed with 1–100 µg/L MC-LR	5 days	Immersion of roots	Proteomics	2-DE gels and MALDI-TOF-TOF MS	Photosynthesis/carbon metabolism and ATP synthesis: PC, ATPα, ATPβ, NADP-MDH, chlorophyll a-b-binding proteins, oxygen-evolving enhancer proteins, quinone oxido-reductase-like protein At1g23740, ATPε, RuBP activase, RuBisCO activase 1, PRK, SBPase, chloroplast Psb04 precursor, Cyt b6f, PsaD, PS II stability/assembly factor HCF136, FNR, γCA2, β-xylosidase/α-L-arabinofuranosidase 2-like, 1-FEH IIa, triosephosphate isomerase, 2,3-bisphosphoglycerate-independent phosphoglycerate mutase, PGK3, ribose-5-phosphate transaldolase, mitochondrial DLST, mitochondrial ATP5δ, PPase1, PDH-E1β, transaldolase-like, chloroplastic soluble inorganic pyrophosphatase 1, ATPγ, chloroplastic-like isoform 1, TK, PFP-β, GADPH, putative cytosolic NADP-malic enzyme, IPMS, SDH, IDH, MDH Stress/defense response: S-formyglutathione hydrolase, IN2-1B, chloroplastic 2-cys peroxiredoxin BAS1, PRX2, TPX, oxidoreductase, CSD2, AKR2, chloroplastic peroxiredoxin-2E, HSP70, PPIase, CPN60α, CPN20, PDI, PDI-L2/3, calreticulin, HSP90, Lea14-A, ClpC, GRXS16, tAPX, TRXR2, PITH domain-containing protein At3g94780, CIpB3, EGS1, HrBP1, and TLP. Protein synthesis and signal transduction: chloroplast putative ribonucleoprotein, cp31-RNP, transcription factor Pur-α 1-like, U2 small nuclear ribonucleoprotein A, zinc finger protein, PCNA, minor allergen Alt a, NACA, EIF3D, elongation factor 1-β, EIF3F, EIF3J, cpRSP1, RPLP0, cpRPL12, PABP, RRAA, EIF5A, 40S ribosomal protein, EIF3K, EEF2, 14-3-3-like protein D-like, 14-3-3-like protein 1-like, and YWHA. Transport activity: TIL, CPP, TIC62, apoD, Ran1A Structural activity: fibrillin, plastid-dividing ring protein, UAM1, and XTH. Other metabolism: KARI, AHAS1, DAPDC2, vitamin b12 independent methionine synthase 5-methyltetrahydropteroyltriglutamate-homocysteine, putative thiosulfate sulfurtransferase, CPOX, GDSL esterase/lipase At5g45670, GDSL esterase/lipase LTL1-like, bifunctional epoxide hydrolase 2-like, ENR1, HACL1-like, PMM, THI1, MIPS, auxin-binding protein ABP20-like, and abscisic acid receptor PYR1-like.	[70]
Male BALB/C mice	0–64 µg CYN/kg bw	Analysis 7 or 14 days after administration	Single i.p. injections	Proteomics	Immunoblotting, SDS-PAGE and ESI-QUAD-TOF	Glomerular integrity: nephrin. Urinary protein profile: serum albumin.	[69]
Marine mussels (*Mytilus galloprovincialis*)	1 × 10^5^ cells/mL CYN-producing *Cylindrospermopsis raciborskii* (equivalent to 7.854 pg/cell of CYN/L) alone or mixed with *Microcystis aeruginosa* (equivalent to 0.023 pg/cell of MC-LR)	15 days	Feeding	Proteomics	FASP, LC-MS/MS	Signaling and communication: RGP51, mec-2, MgC1q12. Cell structure, cytoskeleton and movement: paramyosin, plastin-2, actin, paramyiosin, tropomyosin, α-actin, collagen α-2(I) chain, LCP1, fascin, tubulin β-4B chain Regulation of protein activity: YWHAE, RPN2, cathesin D, CTSB, meprin A subunit α, HSP90, and RPN1. Cell proliferation and migration: ITIH3. Germ cell functions: VEZP9. Energy metabolism: Aldoa, ETFA, and enolase. Gene transcription/translation: RPL30, RPS5, RNA-binding protein, RPL5, and RGN. Cellular calcium ion homeostasis: regucalcin. Embryogenesis: vitellogenin, egg surface protein. Extracellular matrix structure: collagen-like protein-2. Endocytosis: flotillin-1. Regulation of protein activity: arginine kinase. Cellular transport: ARL, MVP, and ATP6V1A. Melatonin biosynthesis: dopamine N-acetyltransferase. Shell structure: nacrein-like protein. Mussel adhesion: byssal protein. Digestive-gland function: trefoil factor.	[68]

Abbreviations: 1-FEH IIa: fructan 1-exohydrolase IIa; 2-DE: Two-dimensional electrophoresis; 4EBP1: Eukaryotic translation initiation factor 4E-binding protein 1; ACAA2: 3-ketoacyl-CoA thiolase mitochondrial; ACC: acetyl-CoA carboxylase; AHAS1: acetohydroxyacid synthase 1; AKR2: aldo-ketoreductase 2-like; AKT: protein kinase B; Aldoa: fructose-bisphosphate aldolase; apoD: apolipoprotein d; Arf: alternate Reading frame; ARL: ADP-ribosylation factor; ASF1A: Anti-silencing function 1B; ATF6: activating transcription factor 6; ATP: adenosine triphosphate; ATP6V1A: V-type proton ATPase catalytic subunit A; BIP: binding immunoglobulin protein; BLMH: Bleomycin hydrolase; bw: body weight; CAT: catalase; CBR1: Carbonyl reductase [NADPH]1; CEP55: centrosomal protein of 55 kDa; CF1: chloroplast-coupling factor 1; CIpB3: chloroplastic chaperone protein; ClpC: chloroplastic chaperone protein; CLPP: ATP-dependent Clp protease proteolytic subunit, mitochondrial; COPG1: coatomer subunit γ-1; cp31-RNP: chloroplast 31 kDa ribonucleoprotein; CPN60α: 60 kDa chaperonin α-subunit; CPOX: chloroplastic coproporphyrigogen-III oxidase; CPP: chloroplast processing peptidase-like; CPN20: chloroplastic 20 kDa chaperonin; cpRPL12: chloroplast 50S ribosomal protein L12; cpRSP1: chloroplast 30S ribosomal protein S1; CSD2: chloroplastic SOD [Cu-Zn]; Cyt b6f: cytochromeb_6_/f heme-binding protein 2-like; DAPDC2: diaminopimelate decarboxylase 2 chloroplastic isoform 1; DLST: dihydrolipoyllysine-residue succinyltransferase component of 2-oxoglutarate dehydrogenase complex 2; EEF2: elongation factor 2; EGS1: eugenol synthase 1; EHMT2: Euchromatic histone lysine methyltransferase 2; EIF2B1: translation initiation factor eIR-2B subunit α; EIF: eukaryotic translation initiation; ENR1: enoyl-ACP reductase 1; EP: extrapallial; ERK1/2: extracelullar signal-regulated kinase; ERP57: 57 kDa endoplasmic reticulum protein; ESI-QUAD-TOF: electrospray ionization quadrupole time-of-flight mass spectrometry; ETFA: electron transfer flavoprotein subunit α; FABP1: fatty acid-binding protein-1; FAM83D: Family with sequence similarity 83 member D; FASN: fatty acid synthase; FASP: filter-aided sample preparation; FERMT2: fermitin family homolog 2; FNR: ferredoxin-NADP reductase; GADPH: glyceraldehyde-3-phosphate dehydrogenase; GPCRs: G protein-coupled receptors; GRP75: 75 kDa glucose regulated protein; GRP78: 78 kDa glucose-regulated protein; GRXS16: glutaredoxin S16; GSHPx: plasma glutathione peroxidase; H: histone; HACL1-like: protein 2-hydroxyacyl-CoA lyase-like; HDFs: human dermal fibroblasts; hESCs: human embryonic stem cells; HrBP1: harpin binding protein 1; HSC71: heat shock cognate 71 kDa isoform 1; HSP60: mitochondrial heat shock 60 kDa protein 1 variant 1; HSP70: heat shock protein 70 kDa isoform 8 variant 2; HSP90: heat shock protein 90α isoform 1; IDH: isocitrate dehydrogenase; IL-6: interleucine-6; IN2-1B: IN2-1 homolog B-like; i.p.: intraperitoneal injection; IPMS: α-isopropylmalate synthase; ITGB1: integrin β-1; ITIH: inter-α-trypsin inhibitor heavy chain; KARI: ketol-acid reductoisomerase; KAT5: Lysine acetyltransferase 5; LC3B: microtubule-associated proteins 1A/1B light chain 3B; LC-MS/MS: Liquid Chromatography-Tandem Mass Spectrometry; LUC7L3: cisplatin resistance-associated overexpressed protein, isoform CRA_b; MALDI-TOF: Matrix-Assisted Laser Desorption/Ionization Time of Flight; MAPK: mitogen-activated protein kinase; MDH: malate dehydrogenase; mec-2: mechanosensory protein 2; MgC1q12: nacre protein and C1q domain containing protein; MIPS: L-myo-inositol-1-phosphate synthase; MMP2: Matrix Metalloproteinase 2; MRP3: multidrug resistance protein 3; mTOR: mammalian target of rapamycin; MVP: major vault protein; MYST1: histone acetyltransferase 1; NACA: nascent polypeptide-associated complex subunit α-like; NADP: nicotinamide adenine dinucleotide phosphate; NuPAGE: new polyacrylamide gel electrophoresis; p16Ink4a: 16 kDa inhibitor of kinase 4, type a protein; p21: Cyclin-dependent kinase inhibitor 1; p21Cip1: 21 kDa CDK-inhibitory protein, type 1; p62: Sequestosome 1; PABP: poly(A)-binding protein; PARP: poly (ADP-ribose) polymerase; PAT: perilipin/ADRP/TIP47 proteins; PC: plastocyanin; PCNA: proliferating cell nuclear antigen; PDH-E1β: pyruvate dehydrogenase E1 component subunit β-like; PDI: protein disulfide isomerase; p-eIF2α: phosphorilation of eukaryotic initiation factor 2 α; pERK1/2: phospho-extracelullar signal-regulated kinase; PFP-β: pyrophosphatase-fructose 6-phosphate 1-phosphotransferase β-subunit; PGK3: phosphoglycerate kinase 3; PGM2: phosphoglucomutase-2; PMM: phosphomannomutase; PPARα: peroxisome proliferator-activated receptor α; PPase1: chloroplastic soluble inorganic pyrophosphatase 1; PPIase: protein peptidyl-propyl cis-trans isomerase; PRK: phosphoribulokinase; PRX2: peroxiredoxin 2; pS2448-mTOR: mammalian target of rapamycin phosphorylated at serine 248; pS473-AKT: protein kinase B phosphorylated at serine 473; PsaD: photosystem I reaction center subunit II; Ran1A: GTP-binding nuclear protein; RANBP2: E3 SUMO-protein ligase RanBP2; RGN: cellular calcium ion homeostasis; RGP51: retrograde protein of 51 kDa; RPL5: 60S ribosomal protein L5; RPL30: ribosomal protein L30; RPLP0: 60S acidic ribosomal protein P0; RPN2: dolichyl-diphosphooligosaccharide-protein glycosyltransferase; RPS5: 40S ribosomal protein S5; RRAA: regulator of ribonuclease activity A; RuBisCO: ribulose bisphosphate carboxylase/oxygenase activase 1; RuBP: ribulose-1,5-bisphosphate carboxylase/oxygenase activase; SAPCD2: suppressor anaphase-promoting complex domain 2; SBPase: sedohetulose-1,7-bisphosphatase; SCD1: stearoyl-CoA desaturase-1; SDH: succinate dehydrogenase; SDS-PAGE: sodium dodecyl sulfate–polyacrylamide gel electrophoresis; GST: glutathione s-transferase protein expression; SOD1: superoxide dismutase 1; SPARC: secreted protein acidic and rich in cysteine; SPINT2: serine peptidase inhibitor, Kunitz type 2; SREBP1: sterol regulatory element-binding protein-1; STUB1: E3 ubiquitin-protein ligase CHIP; SUGT1: protein SGT1 homolog; tAPX: thylakoid-bound ascorbate peroxidase; TCOF1: Treacle protein; TGFβ: Transforming Growth Factor β; THI1: chloroplastic-like thiamine thiazole synthase; TIC62: translocon at the inner chloroplast 62 chloroplastic-like; TIL: temperature-induced lipocalin; TIMP2: Tissue inhibitor of metalloproteinases 2; TK: transketolase; TLP: thaumatin-like protein-like; TNFAIP8: tumor necrosis fator α-induced protein 8; TPX: thioredoxin-dependent peroxidase; TRXR2: thioredoxin reductase 2-like; UAM1: UDP-arabinopyranose mutase 1; VEZP9: vitelline envelope zona pelúcida domain 9; XTH: xyloglucan endotransglucosylase/hydrolase; YWHAE: 14-3-3 protein ε; γCA2: γ carbonic anhydrase-like 2.

### 3.3. Metabolomic: Lipidomic

To date, only two studies have investigated the effects of CYN on lipid profiles. Chowdhury et al. [39] examined CYN-induced disruptions in lipid metabolism using hepatospheroids. Lipid profiling identified 246 lipid species across 19 subclasses and five major lipid categories. While the total lipid abundance did not significantly change, CYN treatment led to an approximately 25% reduction in total protein content and caused notable shifts in lipid composition. Specifically, CYN altered the abundance of certain lipid subclasses and species, such as phosphatidylcholine (PC) and phosphatidylserine (PS). These species were significantly upregulated, alongside marked changes in neutral lipids (including diacylglycerols (DG), triacylglycerols (TG), and cholesteryl esters (CE)), sphingolipids (such as sphingomyelins (SM) or ceramides), and glycerophospholipids (notably lysophosphatidylcholines (LPC), PS, and PC). Additionally, the study revealed that CYN interferes with enzymatic pathways responsible for phospholipid (PL) and sphingolipid interconversion, potentially compromising lipid homeostasis and disrupting hepatic cellular functions.

Casas-Rodríguez et al. [10] investigated the effects of acute exposure to CYN (500 µg/kg bw) on the lipid composition of the rat small intestine. The results revealed specific and time-dependent alterations in several lipid classes, including LPC, SM, PC, PL, and functional heterocyclic compounds over a 24 h period. Notably, LPC levels increased after 6 h of CYN exposure, indicating the activation of inflammatory responses, as LPCs are known as pro-inflammatory mediators. Similarly, SM levels peaked at 4 h and remained elevated, suggesting potential disruption of membrane lipid rafts—specialized domains critical for cellular signaling and structural integrity. In contrast, PC levels decreased in parallel with the LPC increase, pointing to lipid peroxidation and membrane degradation processes likely induced by oxidative stress. These studies demonstrate that CYN can disrupt lipid metabolism and alter lipid profiles in key target organs, including the liver and intestine.

### 3.4. Microbiomic

Saha et al. [71] demonstrated that subchronic oral exposure to CYN (60 µg/kg bw for 15 days) significantly altered the gut microbiota in mice. There was a reduction observed in the abundance of beneficial species, such as *Akkermansia muciniphila*, *Bacteroides thetaiotaomicron*, and *Roseburia_u_s*, and an increase in *Clostridiodes difficile*, an opportunistic pathogen. This dysbiosis was associated with decreased α-diversity and elevated Claudin-2 levels, indicating increased intestinal permeability. The authors also observed liver damage, including inflammation and fibrosis. Therefore, these findings suggest that CYN-induced disruption to the balance of gut microbes contributes to hepatotoxicity through the gut–liver axis.

## 4. Discussion

**Figure 2:**
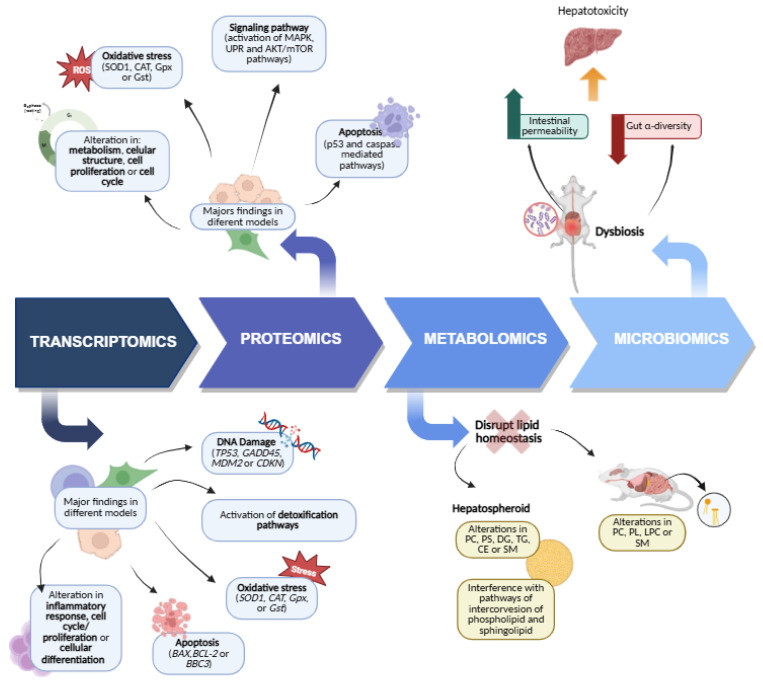
The omics techniques used in the current study on CYN toxicity and mechanisms mainly include transcriptomics, proteomics, metabolomics, and microbiomics. Created with Biorender.com.

Knowledge of CYN toxicity has expanded considerably due to advancements in omics approaches, revealing a complex network of molecular responses across diverse biological systems. Given that CYN has been detected not only in drinking water but also in food sources such as fish, mollusks, and vegetables irrigated with contaminated water, its presence in the food chain represents a growing concern for public health [12]. Despite this, and in contrast to the reviews available for other cyanotoxins such as MCs, no comprehensive review has yet addressed the molecular mechanisms of CYN toxicity from an omics perspective [72]. Therefore, this work reviewed for the first time all the available literature at present using omics approaches to explore the toxicity of CYN in various experimental models that resemble organs from multiple species, providing insights into its potential impact through dietary exposure (Figure 2).

Transcriptomic analyses have been instrumental in providing information on the initial cellular mechanisms of CYN toxicity, particularly in hepatic and renal cells. Most research used PCR-based techniques, with mass sequencing rarely employed. One of the primary mechanisms studied is the metabolism of xenobiotics, which includes detoxification [28,29,30,31,32,33,41,43,48,51]. Thus, a consistent finding about the activation of these pathways is the strong induction of phase I and phase II enzymes, such as CYP1A1 and CYP1A2. It is important to note that while these enzymes often facilitate detoxification, their action can also lead to the bioactivation of compounds, generating more toxic metabolites. Interestingly, 3D hepatic models exhibited even greater induction of these enzymes, which could explain the lower toxicity of CYN in 3D versus 2D cultures and reflect their higher physiological relevance [30]. Specifically, these models showed a pronounced upregulation of genes for both phase I and especially phase II enzymes, which are responsible for the conjugation and elimination of toxic metabolites. However, some studies on differentiated HepaRG cells have noted the down-regulation of xenobiotic metabolism genes, suggesting responses that are specific to the cell type or differentiation state [37]. This highlights that the observed toxicity is not solely dependent on the activation of CYN, but on the balance between metabolic activation and the subsequent detoxification and elimination processes.

DNA damage has been consistently observed in a variety of experimental models, such as hepatic cells [27,28,29,31,32,33], hepatic spheroids [30,39], human embryonic kidney cells [48], intestinal cells [27], dermal fibroblasts [27], human lymphocytes [51], and zebrafish [43]. In general, CYN (0–0.5 µg/mL) does not directly affect *TP53* but impacts downstream genes like *GADD45* and *CDKN*, which are critical for p53 signaling and cell cycle control. Particularly, HepG2 spheroids confirm the induction of DNA damage response genes and cell cycle arrest [30], while differentiated HepaRG cells show a down-regulation of genes involved in cell cycle progression, suggesting a potential growth arrest strategy [37]. CYN also triggers a significant up-regulation of immediate–early response genes (*FOS*, *FOSB*, and *JUNB*) [32]. In different models, like HPBLs, HEK293 cells, and zebrafish, CYN also increases the expression of p53 in a concentration-dependent manner [48,51,62].

The role of CYN on apoptosis is complex. In 2D cultures, although several pro-apoptotic genes are up-regulated, the net effect is often in favor of cell survival due to general down-regulation of BCL2 family members. In contrast, 3D hepatic models clearly show apoptosis induction (via BBC3) [30,36,58]. Moreover, recent findings underscore the importance of ER stress in CYN toxicity. CYN modulates pro-inflammatory gene expression and impacts the regulation of UPR gene biomarkers [38]. This suggests that sustained ER stress and UPR activation are key mechanisms in CYN’s cellular effects, potentially leading to hepatic cell death [34]. Furthermore, at the vascular level, CYN could induce apoptosis by influencing the Rho/ROCK signaling pathway [58].

Different genes related to oxidative stress have been extensively studied, mostly in in vivo models, with a focus on the transcription of antioxidant enzymes such as *SOD*, *GPX*, *GR*, *CAT*, *GST*, *GCLC*, and *H1F1A* (Hypoxia Inducible Factor 1 Alpha), and *Hem* gen which is involved in protoheme synthesis [30,44,45,47,48,51,59,60,62]. CYN is known to induce oxidative stress through the generation of reactive oxygen species (ROS) [73], which correlates with up-regulation of genes like *GPX1*, *GSR*, *GCLC*, and *SOD1* [51]. Conversely, alterations in the gene expression of these oxidative stress-related enzymes can also be linked to an increase in ROS, which in turn might lead to the inhibition of their expression [74].

In relation to liver diseases (steatosis), CYN modulates the expression of genes associated with lipogenesis. RNA sequencing data suggest that CYN induces genes associated with NAFLD, potentially promoting its development and progression in human hepatocytes [34]. Moreover, this finding is supported by results from 3D and in vivo models, which also show that CYN induces lipogenesis and acylglycerol synthesis [39,41,42,43]. In patients with liver cancer, there is a positive correlation between CYN serum levels and the expression of genes involved in PPAR signaling and lipid metabolism. In this sense, there is evidence of a link between cyanotoxins and the pathogenesis of hepatocellular carcinoma and the progression of hepatic steatosis [46]. Compared with other cyanotoxins, such as MCs, KEGG pathway enrichment revealed that MC-LR exposure activated some important signaling pathways, including cancer-related pathways, the PI3K-AKT signaling pathway, and the MAPK signaling pathway [75]. Moreover, PPARα is a key target of hepatic steatosis and saturated fatty acid accumulation induced by MC-LR in zebrafish [76].

Additionally, CYN also affects other genes involved in a variety of processes, including inflammatory response [52,54], cell cycle/proliferation, tubular transport processes (affecting key genes involved in endocytosis and ionic homeostasis) [49], RNA processing [37], cellular differentiation [56], cytoskeleton organization [58], and neuronal development [57].

Though proteomic studies are fewer in number, they are more varied in terms of techniques, which complement and confirm transcriptomic findings, offering direct insights into cellular protein changes.

Regarding the liver, CYN upregulates antioxidant defense proteins like *GPX*, *SOD1*, *CAT*, *GST*, and *MRP3* [34,45,47,65]. In different cases, the relative abundance of the same enzymatic antioxidant proteins did not correlate with the activity and the gene expression of the enzyme, suggesting that there is a regulation at the transcriptional and translational level or post-translational modifications. Changes in the level of the GST enzyme were also observed in the kidney [45,47]. It should be noted that in bivalves exposed to *C. raciborskii*, which produces CYN, a decrease in HSP60 protein associated with a stress response was also observed [67]. These findings confirm that CYN causes an oxidative stress response at the protein level.

Regarding apoptosis, CYN can activate p53-dependent (increased p53) and caspase-mediated pathways (PARP cleavage, caspase-3 activation) [27,36], making it clear that intrinsic and extrinsic apoptotic pathways could be implicated.

Other observed changes include disruptions in energy metabolism (upregulated glycolysis proteins) and changes in cytoskeleton structure [65,67], cellular metabolism and regulation, and synthesis and transport of proteins [48,66].

In liver stem cells, CYN triggers MAPK signaling activation [38]. Moreover, CYN’s effect on protein folding is mixed: some chaperones decrease (HSP70, GRP75), while others increase (HSC71, HSP90, and GRP78), indicating UPR activation [65]. CYN promotes hepatic steatosis by elevating proteins involved in lipid metabolism (acetyl-CoA carboxylase, stearoyl-CoA desaturase-1, prosaposin, and ACAA2 (3-ketoacyl-CoA thiolase mitochondrial)) and potentially impairing autophagy [34,39,48]. However, the expression of the four fibrosis-signaling protein markers was not significantly modulated by CYN. Considering the MAPK, UPR, and AKT/mTOR pathways, their involvement is confirmed at both the transcriptional and protein levels.

Studies have demonstrated that CYN affects pathways involving proteins crucial for cell proliferation, cell cycle regulation, and cytokinesis. Specifically, CYN has been shown to interact with key cell cycle checkpoints, such as p16 and p21, as well as various cyclins [66]. This interaction is particularly significant because precise control of cell division is vital for life. When this control is disrupted or dysregulated, it can lead to serious consequences, notably being a hallmark of cancer. In this sense, in hepatic cells, it was demonstrated that CYN altered protein pathways.

On the other hand, metabolomics is key to understanding how metabolic processes influence cancer progression. By studying metabolites, which are direct indicators of disease, this discipline allows us to decipher the relationship between physiological states, external factors, and disease development. Its ability to offer a comprehensive molecular analysis positions metabolomics as a decisive tool in precision medicine [77]. In this sense, the few existing metabolomics studies with CYN are mainly focused on the lipid profile [10,39]. These studies provide compelling evidence that CYN exposure disrupts lipid homeostasis in different organ systems through distinct but interconnected mechanisms. Together, these findings highlight CYN’s capacity to disrupt cellular lipid architecture and signaling, contributing to toxicological outcomes in both hepatic and intestinal tissues. Other cyanotoxins have also shown effects on lipid metabolism. Thus, MC-LR was found to promote the development of NASH by disrupting the lipid metabolic function. Furthermore, MC-LR impacted various metabolic pathways within the liver. Specifically, it affected tyrosine metabolism, choline metabolism, energy metabolism, nucleotide synthesis, and glutathione detoxification [78]. All of this highlights the need for further research into CYN metabolomics.

Finally, microbiome research reveals that subchronic oral CYN exposure in mice significantly alters gut microbiota composition. This includes a reduction in beneficial species and an increase in opportunistic pathogens. This dysbiosis is associated with decreased gut α-diversity and increased intestinal permeability, ultimately contributing to hepatotoxicity via the gut–liver axis [71]. In view of all this, more research is necessary to obtain a better understanding of how CYN affects the gut microbiota and its role in metabolism, especially given that other cyanotoxins, such as MC-LR, have been shown to disturb microbial species involved in lipid and energy metabolism, as revealed by Lin et al. [79].

**Figure 3:**
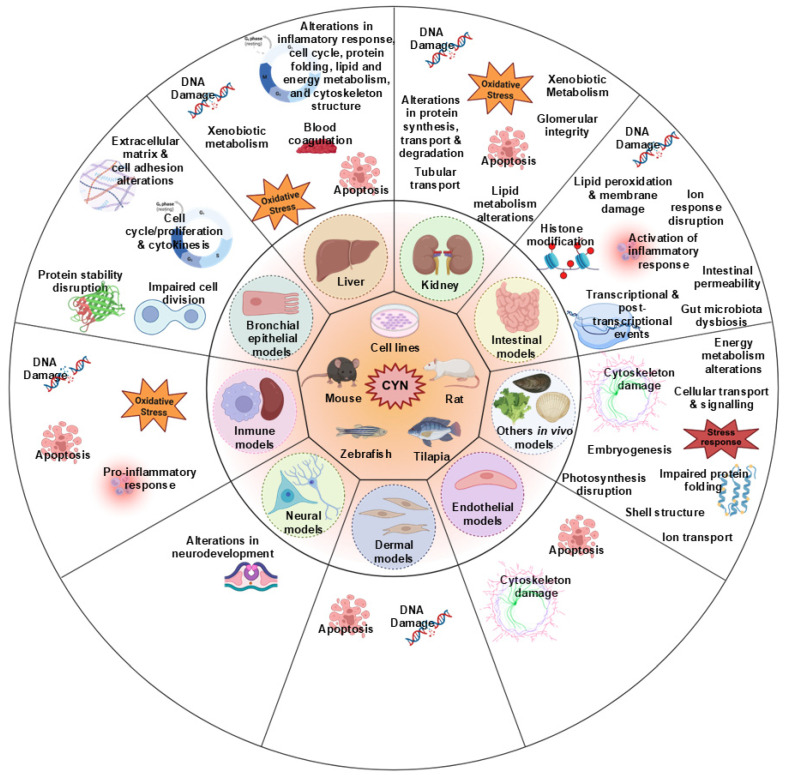
Omics techniques revealed multi-organ toxicity and possible mechanisms induced by CYN. The current omics data showed that CYN could exert toxic effects on multiple organs, including liver, kidney, intestinal, bronchial, epithelial, immune, neural, endothelial, and dermal models, and other systems. The main adverse effects include oxidative stress, apoptosis, and DNA damage, as well as metabolism alteration. Created with Biorender.com.

The application of omics techniques has illuminated the multi-organ effects and possible modes of action for CYN (Figure 3). Despite significant advances, current omics research on CYN toxicity reveals notable gaps. There is a particular limitation in understanding its impact on the immune system, neurotoxicity, reproductive toxicity, and the pancreas. Mainly, the absence of mass sequencing at the transcriptomic level hinders comprehensive insights into gene expression changes and the implication of fibrosis signaling.

Taken together, the molecular alterations induced by CYN indicate potential adverse effects in humans that could compromise organ function. The activation of oxidative stress and DNA damage pathways suggests the genotoxic potential of CYN, while alterations in lipid metabolism and protein folding may contribute to hepatic steatosis or fibrosis. Similarly, effects on tubular transport, immune response, and neurological development raise concerns about systemic toxicity and the risks of long-term exposure. Therefore, the integration of omics data is essential to better assess the risk that exposure to CYN may pose to human health.

Moreover, the actual risk associated with exposure to CYN through contaminated food remains uncertain due to analytical limitations. Matrix effects, particularly in complex food and environmental samples, can significantly affect the sensitivity and accuracy of determinations, leading to either under- or over-estimation of toxin concentrations [19]. These effects are not always systematically evaluated, and their impact can vary widely depending on the toxin–matrix combination, further complicating reliable quantification [19]. Moreover, the co-occurrence of CYN with other cyanotoxins or structurally related compounds may further compromise analytical reliability [80].

In addition, a key gap remains in our understanding of the toxicokinetics of CYN, particularly about how the toxin is metabolized once inside the organism. While it has been reported that CYP450 enzymes are involved in CYN-induced toxicity, the exact metabolites produced by these biotransformation pathways have yet to be identified in vivo [6]. Evidence from aquatic models indicates that CYN can disrupt fundamental detoxification mechanisms. However, the magnitude and nature of the reported responses vary considerably across experimental species and models. According to EFSA [19], this variability likely reflects methodological differences, such as route, duration, and dose of exposure. Many cyanotoxin studies still rely on intraperitoneal administration, which produces higher toxicity than oral exposure, which is the most relevant for humans. While most of the quantitative data come from microcystins, the EFSA emphasized that the available information for CYN remains scarce and insufficient to derive robust health-based guidance values, underscoring the need for standardized exposure and toxicokinetic data. These methodological and data limitations complicate the interpretation of the experimental findings and increase uncertainty when extrapolating results to humans.

These findings highlight the importance of conducting comprehensive metabolomic and lipidomic profiling in species relevant to the food chain, in order to clarify the systemic metabolic alterations induced by CYN. Therefore, future research must prioritize the development of multi-analyte methods capable of compensating for these analytical issues, alongside genomics, metabolomics, and microbiomics, to fully elucidate CYN’s systemic effects and strengthen the evidence base for dietary risk assessment. Ultimately, a thorough understanding of CYN’s risks is essential for developing effective strategies to protect consumer health.

Our work contributes to the integration of omics-based data within the new approach methodologies (NAMs) framework, which the EFSA Strategy 2027 strongly encourages for food-related risk assessment [81].

## 5. Conclusions

CYN is a complex, systemic cyanotoxin, as evidenced by the profound alterations observed at multiple ‘omic’ levels. At the transcriptomic level, CYN modulates gene expression, affecting processes such as detoxification, DNA damage, ER stress/UPR, oxidative stress, and lipogenesis. This suggests a potential role in the development of NAFLD. At the proteomic level, these changes result in alterations to the antioxidant defense system, protein folding, lipid metabolism, apoptosis, and cytoskeletal integrity. The response varies depending on the experimental model and concentration/dose. Furthermore, the activation of key pathways such as MAPK, UPR, and AKT/mTOR was confirmed by both transcriptomic and proteomic analysis. Although research in the areas of lipidomics and microbiomics is limited, CYN has been shown to disrupt lipid homeostasis by affecting hepatic metabolic pathways and inducing gut dysbiosis by reducing beneficial species and increasing pathogens. This impacts the intestinal barrier and contributes to hepatotoxicity. Overall, further advances and research in transcriptomics, proteomics, lipidomics, and microbiomics are needed. This comprehensive approach is essential to understand the hazardous profile of CYN, given its potential presence in food, and therefore, its risks and consequences for human health.

## Data Availability

No new data were created or analyzed in this study. All information was obtained from the previously published sources cited in the article.

## Abbreviations

The following abbreviations are used in this manuscript:

CYN	Cylindrospermopsin
MCs	Microcystins
CYP450	Cytochrome P450
AChE	Acetylcholinesterase
WHO	World Health Organization
TDI	Tolerable daily intake
Bw	Body weight
EFSA	European Food Safety Authority
CYP1A1	Family 1, subfamily A, polypeptide 1
CYP1A2	Cytochrome P450, family 1, subfamily A, polypeptide 2
*TP53*	Tumor Protein p53
*GADD45*	Growth arrest and DNA-damage-inducible
*CDKN1A*	Cyclin-Dependent Kinase Inhibitor 1A
*FOS*	FBJ murine osteosarcoma viral oncogene homolog
*FOSB*	FBJ murine osteosarcoma viral oncogene homolog B
*JUNB*	Jun B pro-to-oncogene
UPR	Unfolded protein response
ER	Endoplasmic reticulum
RT-qPCR	Reverse transcription quantitative polymerase chain reaction
NAFLD	Non-alcoholic fatty liver disease
hESCs	Human embryonic stem cells
*FOXA2*	Forkhead box A2
*AFP*	Alpha fetoprotein
*CX43*	Gap Junction Protein Alpha 1
*ALB*	Albumin
*TTR*	Transthyretin
*CX32*	Gap Junction Protein Beta 1
LSCs	Liver stem cells
*HSPA5*	Heat Shock Protein Family A (Hsp70) Member 5
*ATF3*	Activating Transcription Factor 3
*NAT1*	N-Acetyltransferase 1
*NAT2*	N-Acetyltransferase 2
*SULT1B1*	Sulfotransferase Family 1B Member 1
*SULT1C2*	Sulfotransferase Family 1C Member 2
*UGT1A1*	UDP Glucuronosyltransferase Family 1 Member A1
*UGT2B7*	UDP Glucuronosyltransferase Family 2 Member B7
*BBC3*	BCL2 Binding Component 3
*SREBF1*	Sterol regulatory element-binding protein 1
*DGAT1/2*	Diacylglycerol O-acyltransferase 1/2
i.p.	Intraperitoneal
Nrf2	Nuclear factor erythroid 2-related factor 2
*Fabp4*	Fatty acid-binding protein 4
NASH	Non-alcoholic steatohepatitis
*PPAR*	Peroxisome proliferator-activated receptor
Gpx	Glutathione peroxidase
Gst	Glutathione-S-transferase
*CAT*	Catalase
*SOD1*	Superoxide Dismutase 1
*dab2*	DAB Adaptor Protein 2
*ATP1A1*	ATPase Na+/K+ transporting subunit alpha 1
*BCL2*	B-cell CLL/lymphoma 2
*BAX*	Bcl2-Associated X protein
HPBLs	Human peripheral blood lymphocytes
*MDM2*	Human homologue of mouse double minute 2
*GSR*	Glutathione-disulfide reductase
*GCLC*	Glutamate-cysteine ligase catalytic subunit
*IL-2*	Interleukin 2
*IL-6*	Interleukin 6
*IFN-γ*	Interferon Gamma
*TNF-α*	Tumor Necrosis Factor
*IL-1β*	Interleukin 1 Beta
*IL-10*	Interleukin 10
*TGF-β*	Transforming Growth Factor Beta 1
mES	Mouse embryonic stem
EBs	Embryoid bodies
*NTNG2*	Netrin G2
*KCNJ11*	Potassium Inwardly Rectifying Channel Subfamily J Member 11
*SLC18A3*	Solute Carrier Family 18 Member A3
*APOE*	Apolipoprotein E
*SEMA6B*	Semaphorin 6B
HDFs	Human dermal fibroblasts
HUVECs	Human umbilical vein endothelial cells
*ITGB1*	Integrin β-1
*Rho*	Rhodopsin
*ROCK*	Rho-associated coiled-coil containing protein kinase 1
*MLC-1*	Megalencephalic leukoencephalopathy with subcortical cysts 1
*VIM-1*	Vimentin
*prkg1*	Protein kinase cGMP-dependent 1
*gucy1A1*	Guanylate cyclase 1 soluble subunit alpha 1
*akt*	Serine/threonine-protein kinase
ALP	Alkaline phosphatase
*leuB*	3-isopropylmalate dehydrogenase
*sds*	Serine dehydratase
phoD	Alkaline phosphatase
sec1β	SEC61 translocon subunit beta
*fshr*	Follicle-stimulating hormone receptor
*lhr*	Luteinizing hormone/choriogonadotropin receptor
*igf3*	Insulin-like growth factor 3
hCG	Human chorionic gonadotropin
*srd5a2*	Steroid-5-alpha-reductase, alpha polypeptide 2a
*3βhsd*	Hydroxy-delta-5-steroid dehydrogenase, 3 beta- and steroid delta-isomerase 2
*cyp19a1a*	Cytochrome p450 family 19 subfamily A polypeptide 1a
*cyp11a2*	Cytochrome p450 family 11 subfamily A member 2
*hsd17b1*	Hydroxysteroid (17-beta) dehydrogenase 1
*hsd17b3*	Hydroxysteroid (17-beta) dehydrogenase 3
*aldob*	Aldolase
*pgm2*	Phosphoglucomutase 2
*g6pca.2*	Glucose-6-phosphatase catalytic subunit 1a, tandem duplicate 2
*lamb1a*	Laminin beta 1a
*col4a5*	Collagen type IV alpha 5 chain
*itga3b*	Integrin alpha 3b
MRP3	Multidrug resistance protein 3
HSP70	Heat shock protein 70 kDa isoform 8 variant 2
GRP75	75 kDa glucose-regulated protein
HSC71	Heat shock cognate 71 kDa isoform 1
HSP90	Heat shock protein 90α isoform 1
GRP78	78 kDa glucose-regulated protein
HSP60	Mitochondrial heat shock 60 kDa protein 1 variant 1
ACC	Acetyl-CoA carboxylase
SCD1	Stearoyl-CoA desaturase-1
SREBP-1	Sterol regulatory element-binding protein-1
FABP1	Fatty acid-binding protein-1
FASN	Fatty Acid Synthase
PPARα	Peroxisome Proliferator-Activated Receptor alpha
ADRP	Perilipin 2
MAPK	Mitogen-activated protein kinase
ERK1/2	Extracellular signal-regulated kinase 1/2
KAT5	Lysine acetyltransferase 5
MYST1	Histone acetyltransferase 1
EHMT2	Euchromatic histone lysine methyltransferase 2
SPINT2	Serine peptidase inhibitor, Kunitz type 2
SPARC	Secreted protein acidic and rich in cysteine
Agrin	APLNR
FAM83D	Family with sequence similarity 83 member D
SAPCD2	Suppressor anaphase-promoting complex domain 2
CEP55	Centrosomal protein of 55 kDa
EP	Extrapallial fluid protein
PC	Phosphatidylcholine
PS	Phosphatidylserine
DG	Diacylglycerols
TG	Triacylglycerols
CE	Cholesteryl esters
SM	Sphingomyelins
LPC	Lysophosphatidylcholines
PL	Phospholipid
H1F1A	Hypoxia Inducible Factor 1 Alpha
ROS	Reactive oxygen species
ACAA2	3-ketoacyl-CoA thiolase mitochondrial
